# Digital sequencing is improved by using structured unique molecular identifiers

**DOI:** 10.1186/s13059-025-03504-x

**Published:** 2025-02-25

**Authors:** Peter Micallef, Manuel Luna Santamaría, Mandy Escobar, Daniel Andersson, Tobias Österlund, Pia Mouhanna, Stefan Filges, Gustav Johansson, Henrik Fagman, Christoffer Vannas, Anders Ståhlberg

**Affiliations:** 1 Department of Laboratory Medicine, Institute of Biomedicine, Sahlgrenska Center for Cancer Research, Sahlgrenska Academy, University of Gothenburg, Gothenburg, 413 90 Sweden; 2 Department of Clinical Genetics and Genomics, Sahlgrenska University Hospital, Gothenburg, 413 45 Sweden; 3 Wallenberg Centre for Molecular and Translational Medicine, University of Gothenburg, Gothenburg, 413 90 Sweden; 4 Department of Oncology, Ryhov County Hospital, Jönköping, 551 85 Sweden; 5 Simsen Diagnostics AB, 411 26 Gothenburg, Sweden; 6 Department of Clinical Pathology, Sahlgrenska University Hospital, Gothenburg, 413 45 Sweden; 7 Department of Oncology, Sahlgrenska University Hospital, Gothenburg, 413 45 Sweden

**Keywords:** Digital sequencing, Error-free sequencing, Molecular barcode, Sequencing, Unique molecular identifier

## Abstract

Digital sequencing uses unique molecular identifiers (UMIs) to correct for polymerase induced errors and amplification biases. Here, we design 19 different structured UMIs to minimize the capacity of primers to form non-specific PCR products during library construction using SiMSen-Seq, a PCR-based digital sequencing approach with flexible multiplexing capabilities suitable for tumor-informed mutation analysis. All structured UMI designs demonstrate enhanced assay performance compared with an unstructured reference UMI. The best performing structured UMI design shows significant improvements in all tested aspects of assay and sequencing performance with the ability to reliable detect low variant allele frequencies.

**Supplementary Information:**

The online version contains supplementary material available at 10.1186/s13059-025-03504-x.

## Background

Massively parallel sequencing enables a growing number of clinical and basic research applications within many diverse areas, including diagnostics, treatment stratification, drug discovery, forensics, evolutionary studies, and environmental DNA testing. Essentially, any type of biological sample can be analyzed, comprising complex sample matrices, such as tissues, body fluids, and environmental samples. Biological samples may be of highly variable sample sizes, ranging from individual molecules to billions of cells. The DNA itself may be of diverging integrity, e.g., intact DNA extracted from living cells or highly fragmented DNA that is typical for fixed tissues, body fluids, and forensic samples. Numerous technical approaches exist to analyze variable amounts of sequences, ranging from a handful of loci to entire genomes. However, conventional sequencing techniques can only detect variant allele frequencies down to 1–5% [1–3]. This is insufficient for several emerging applications, such as circulating tumor-DNA (ctDNA) analysis, requiring reliable detection of variant allele frequencies < 0.1% or even individual molecules [4, 5].

To overcome this issue, unique molecular identifiers (UMIs), also known as molecular barcodes, are introduced in library construction to enable digital sequencing [6–8]. The UMI, which typically consists of an 8–12 nucleotides long randomized sequence, is used to label target DNA using either PCR- or hybridization capture-based approaches. There are at least 25 digital sequencing approaches, even excluding all updated versions of specific strategies and commercialized methods [9]. The PCR-based strategies are normally applied to small- and medium-sized panels (10^2^–10^5^ nucleotides), while hybridization capture-based approaches are mostly used for medium- to large-sized panels (10^4^ to 10^6^ nucleotides). SiMSen-Seq [10] and Safe-SeqS [6] are examples of PCR-based strategies and variants of duplex sequencing [7, 11–13] are examples of hybridization capture-based approaches. All sequence reads with identical UMI can be bioinformatically traced back to the same original template DNA molecule, generating consensus reads that enable correction of polymerase-induced errors and minimization of quantification biases (Additional file 1: Fig. S1) [4]. However, as the random sequence of UMIs is prone to generate non-specific PCR products that interfere with the overall performance of library construction, especially in PCR-based approaches, most experimental protocols tend to be complicated, consisting of several experimental steps. Experimental protocols that mitigate the shortcomings of UMIs will facilitate the implementation of digital sequencing in clinical and basic research [9].

We hypothesized that the formation of non-specific PCR products caused by UMIs could be reduced by inserting predefined nucleotides at specific positions within the UMIs, i.e., structuring the UMIs to reduce the possibility of stable and unwanted interactions both within as well as between molecules, while maintaining high PCR efficiencies and sensitivity. Here, we developed an improved digital sequencing approach using structured UMIs based on SiMSen-Seq (simple multiplexed PCR-based barcoding of DNA for ultrasensitive mutation detection using next-generation sequencing) [14]. The performance of 19 different structured UMI strategies was evaluated using quantitative PCR, parallel capillary electrophoresis, melting curve analysis, and sequencing. We show experimentally how specificity and sensitivity of both individual assays and multiplexed panels are universally improved using structured UMIs. Table 1 shows the applied metrics used to assess different UMI designs. Finally, we demonstrate utility with the best performing approach by assessing low variant allele frequencies in standardized control material as well as monitoring ctDNA levels in blood plasma collected from a patient diagnosed with leiomyosarcoma.

**Table 1:** Metrics to evaluate different UMI designs

Method	Tested parameter
Quantitative PCR	Assay specificity (ΔCq)
Parallel capillary electrophoresis	Library purity (%)
Melting curve analysis	Stem-loop stability (°C)
Digital sequencing	Number of detected molecules
Digital sequencing	Off-target reads (%)
Digital sequencing	Number of mutated molecules
Digital sequencing	Variant allele frequency (%)
Digital sequencing	Error rate (%)

## Results

### Design of different UMI structures in digital sequencing

**Fig. 1:**
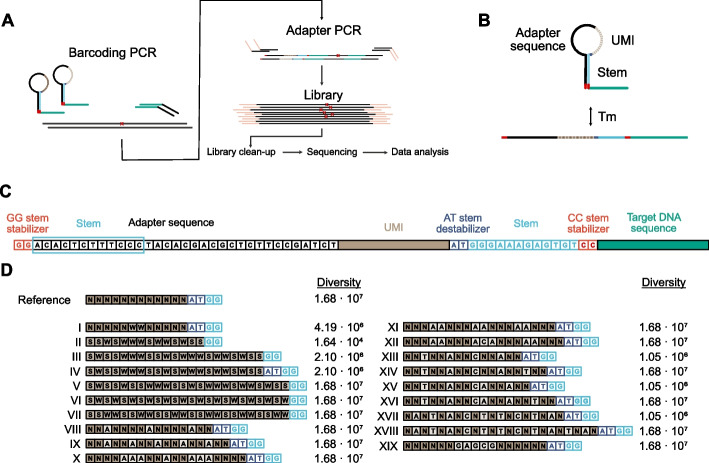
Digital sequencing using structured UMIs. **A** Overview of the SiMSen-Seq workflow. In SiMSen-Seq, each double-stranded target DNA molecule generates on average two different UMIs after three cycles barcoding PCR and a threefold dilution before adapter PCR [53]. **B** Schematic structure and function of SiMSen-Seq barcode primers with stem-loop protected UMI. Stem opening is temperature dependent. Tm, melting temperature. **C** Design and sequence of forward SiMSen-Seq barcoding primers. Different primer elements are indicated by color and name. Blue box indicates the part of the adapter sequence that complementary hybridizes to the blue sequence to form the stem. The stem is stabilized by two nucleotide pairs (GG and CC stem stabilizers) and destabilized by two nucleotides (AT stem destabilizer). The AT stem destabilizer prevents the UMI from extending the stem length. **D** Design of structured UMIs. The sequence contexts of 19 structured UMIs, I–XIX, and the unstructured reference UMI are shown (for additional details, see Additional file 3: Table S4). Designs II, III, V, VI, and VII lack the AT stem destabilizer. Nucleotide N represents any nucleotide type; nucleotide S represents cytosine or guanine; nucleotide W represents adenine or thymine

To test our hypothesis that structured UMIs form less unintended primer interactions reducing the formation of non-specific PCR products during library construction, we used SiMSen-Seq. The protocol consists of two sequential rounds of PCR, i.e., barcoding and adapter PCR, followed by a single purification step before sequencing (Fig. 1A). In the barcoding PCR step, primers containing UMIs are used to label target DNA. In the subsequent adapter PCR step, barcoded target DNA is amplified using sequencing adapter primers. The rationale of using SiMSen-Seq is that this approach already utilizes three strategies that all on their own reduce generation of non-specific PCR products, i.e., (i) protecting the UMI in a stem-loop structure that is closed during the primer annealing step of barcoding PCR, while open during the primer annealing step of the adapter PCR (Fig. 1B), (ii) limiting the primer concentration in the barcoding PCR, and (iii) terminating the barcoding PCR by an inactivation buffer containing protease.

We designed and evaluated 19 different UMI structures, I–XIX (Fig. 1C–D), based on different hypotheses, aiming to reduce the capacity of primers to form undesirable internal structures and interactions with other primers or input DNA (Additional file 1: Fig. S2). At the same time, structured UMIs need to perform experimentally well in the SiMSen-Seq protocol. The performance of each design was compared with an unstructured reference UMI, consisting of a conventional 12 nucleotides long randomized sequence. Seven designs (I–VII) utilized different combinations of degenerated nucleotides to reduce the risk of forming G-quadruplexes and other unintended internal stem structures. Sequences with balanced GC and AT content usually perform well with high PCR efficiencies. Twelve designs (VIII–XIX) divided the UMIs into smaller segments of randomized nucleotides. Four of these designs (VIII–XI) used variable numbers and positions of adenine. The rationale of using only adenine as structured nucleotides is twofold: (i) any unintended internal sequence interactions are inherently less likely compared to a combination of adenine and thymine, and (ii) any interactions are thermodynamically weaker in comparison to adenine in any combination with cytosine and guanine. The potential drawback with these designs is that they may generate longer homopolymers with only adenines that may perform less efficiently in library construction and sequencing. One design (XII) used a combination of adenine and cytosine. Six designs (XIII–XVIII) used different combinations of adenine, cytosine, and thymine. One design (XIX) used a UMI structure with a combination of five nucleotides that are complementary to an internal sequence in the adapter sequence between the stem and UMI (Additional file 1: Fig. S2B). The concept with this design was to test if an even stronger stem could reduce generation of non-specific PCR products. The number of possible UMI combinations for a given design, i.e., UMI diversity, is defined by the total UMI length and number of alternative nucleotides allowed at each position (Fig. 1D). High UMI diversity is required to reduce the risk that two target DNA molecules are labeled with identical UMI. The risk of UMI collision for the designs with highest and lowest diversity is outlined in Additional file 1: Fig. S3. Thirteen designs, including the unstructured reference UMI, displayed a diversity of 16.8 million combinations. The other designs consisted of less randomized nucleotides and hence lower diversity, where designs XIII, XV, and XVII displayed a diversity of 1.05 million combinations, design I 4.19 million combinations, designs III and IV 2.10 million combinations, and design II 16,400 combinations. To estimate the amount of UMI-UMI interactions that may occur in relation to all different UMI designs, we performed a simulation (Additional file 1: Fig. S4). Here, UMI designs I, VIII–XII, XV, and XVII generated lower amounts of UMI-UMI interactions compared with unstructured reference UMI, while the other structured UMI designs resulted in more UMI-UMI interactions.

### Structured UMIs improve assay performance

**Fig. 2:**
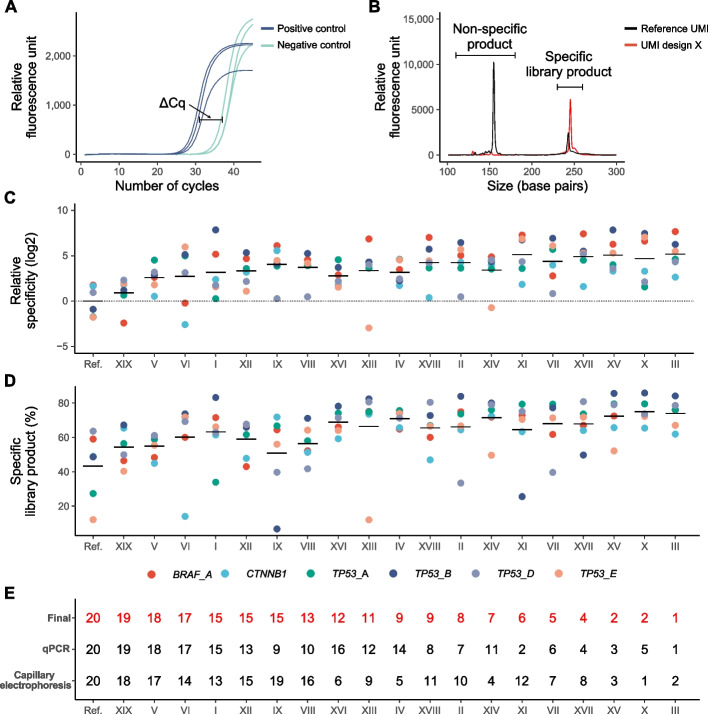
Assay performance of different structured UMI designs. **A** Assay performance based on quantitative PCR. The relative specificity is indicated as ΔCq, calculated as the difference in cycle of quantification values between samples with 20 ng DNA (blue) and without DNA (turquoise). ΔCq equals one corresponds to a twofold difference in assay specificity, assuming 100% PCR efficiency. The figure shows the amplification curves of a representative assay (*TP53_A*, UMI design X, *n* = 3). **B** Assay performance based on parallel capillary electrophoresis. The black and red electropherograms exemplify representative libraries for a representative assay (*TP53_A*) with reference UMI and UMI design X, respectively, using 20 ng DNA, *n* = 1. **C** Relative specificity for all UMI designs based on quantitative PCR. Mean value for each individual assay is shown, *n* = 3. The mean of all assays is indicated by a bar for each individual UMI design. Data are normalized to the reference UMI, which is mean-centered. **D** Specificity for all UMI designs based on correct library product formation using parallel capillary electrophoresis. The percentage of specific library products relative total DNA amount is shown for six assays (*n* = 1) with mean indicated by a bar. **E** The final rank of all the different UMI designs based on their relative performance using both quantitative PCR (qPCR) and parallel capillary electrophoresis data. The order of UMI designs in **C** and **D** are based on their final ranks

We performed two tests to determine assay specificity of structured UMI designs. In the first approach, we carried out the adapter PCR in the library construction protocol as a quantitative PCR assay (Fig. 2A). To compare the relative specificity between different structured UMIs, we determined the differences in cycle of quantification values between DNA positive and negative samples, where a large difference indicate superior performance. In the second test, we evaluated library purity in unpurified libraries using parallel capillary electrophoresis (Fig. 2B). We assessed specificity as the amount of specific library relative to total amount of DNA. The performance of each design was compared with the unstructured reference UMI.

We used six different SiMSen-Seq assays to analyze 20 ng genomic DNA per reaction, using water as negative control, with quantitative PCR. Overall, all structured UMI designs displayed improved specificity compared to the reference UMI (Fig. 2C). Design III performed best with 36 times higher specificity than reference UMI, followed by designs XI, XV, XVII, and X. Next, we evaluated assay performance on final library purity in samples generated from 20 ng genomic DNA with parallel capillary electrophoresis (Fig. 2D and Additional file 1: Fig. S5). Again, all structured UMI designs were superior to the unstructured reference UMI. UMI design X performed best with 32 percentage points more specific library products compared with reference UMI (75% versus 43%), followed by UMI designs III, XV, XIV, and IV.

To summarize the overall performance of different structured UMI designs, we ranked them in each evaluation test and calculated their total ranking score, providing a final rank list (Fig. 2E). Structured UMI design III demonstrated highest specificity followed by UMI designs X, XV, XVII, and VII. UMI designs with lower UMI diversity showed no overall improvements in assay specificity compared to designs with high UMI diversity. However, closely related UMI designs systematically performed somewhat better with less randomized nucleotides, e.g., design III versus V, design XV versus XVI, and design XVII versus XVIII, while designs XIII and XIV performed similarly. Interestingly, the performance of the individual UMI designs could not be explained by their theoretical ability to form UMI-UMI interactions (Additional file 1: Fig. S6), indicating that other types of interactions caused the formation of non-specific PCR products. We selected UMI design X for further downstream analysis, since it displayed the highest specificity of all designs with the highest diversity.

An important feature in SiMSen-Seq primer design is to maintain the melting temperature of the stem structure above the primer annealing temperature used in the barcoding PCR and below the primer annealing temperature of adapter PCR (Fig. 1A–B). High resolution melting curve analysis of forward barcoding PCR primers showed that most structured UMI designs displayed somewhat lower melting temperatures compared with unstructured reference UMI (Additional file 1: Fig. S7A–B). The exception was UMI design XIX that showed a higher melting temperature, which was expected since this design contained an extra internal stem sequence. The melting temperature of each individual UMI design was significantly inversely correlated with nucleotide length of the loop structure (Additional file 1: Fig. 7C). However, the melting temperature remained > 7 °C above the annealing temperature used in the barcoding PCR, thus ensuring a closed stem structure.

### Validation of improved assay performance using structured UMIs

**Fig. 3:**
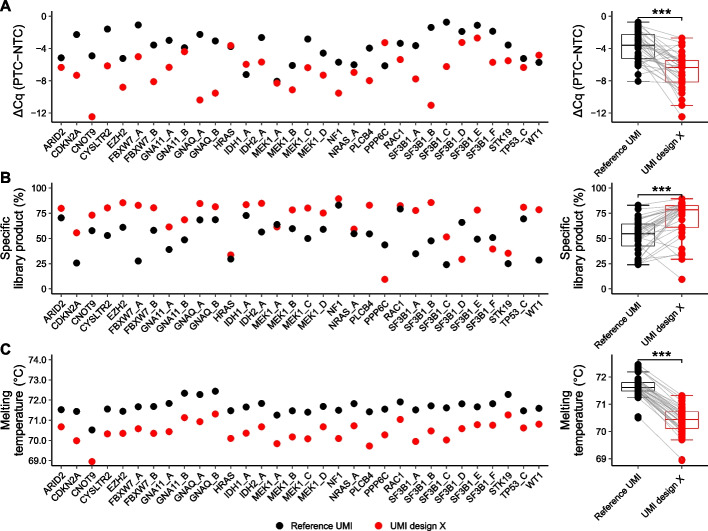
Validation of improved assay performance using structured UMIs. Data for UMI design X and reference UMI are shown for 32 individual assays using 20 ng DNA. **A** Relative specificity using quantitative PCR. Mean ΔCq was calculated as the difference in cycle of quantification values between samples with DNA (PTC) and no template control (NTC). Mean value is shown for each assay and UMI design, *n* = 3. Box plots of all data are shown to the right. ***p ≤ 0.001, Wilcoxon signed-rank test, *n* = 32. **B** Specificity based on correct library product formation using parallel capillary electrophoresis. The percentage of specific library products relative total DNA amount is shown, *n* = 1. Box plots of all data are shown to the right. ***p ≤ 0.001, Wilcoxon signed-rank test, *n* = 32. **C** Melting temperatures of forward barcoding primers. Mean melting temperature is shown for each assay and both UMI designs, *n* = 2. Box plots of all data are shown to the right. ***p ≤ 0.001, Wilcoxon signed-rank test, *n* = 32

In addition to UMI design, assay performance is also dependent on target DNA sequence of each primer. To further verify that structured UMIs are superior to the unstructured reference UMI, we designed and tested 32 additional assays. We used UMI design X in the benchmarking and again tested assay performance using quantitative PCR and parallel capillary electrophoresis. Figure 3A shows that 28 of 32 assays displayed improved assay specificity with structured UMI design X when compared with reference UMI using quantitative PCR. The mean improvement in relative specificity was 8.0 times. We observed no correlations between assay performance and the ability for target primer sequences to form homo- or hetero-dimers (Additional file 1: Fig. S8A–C) nor to GC content in the forward target primer (Additional file 1: Fig. S8D). The specificity in library construction based on parallel capillary electrophoresis was also improved in 28 of 32 assays with a mean improvement from 52 to 69% in specific library product formation (Fig. 3B). Out of these 28 assays, 25 had also demonstrated superior performance in quantitative PCR. One assay, *PPP6C*, performed poorly for UMI design X using both quantitative PCR and parallel capillary electrophoresis.

We also determined the stem melting temperature for all 32 assays (Fig. 3C). All assays displayed a narrow range of melting temperatures between 69 and 73 °C, indicating functional stem-loop structures. UMI design X decreased the mean melting temperature with 1.2 °C. Interestingly, we observed a correlation in melting temperature of the stem-loop between the assays regardless of UMI structure (Spearman’s correlation coefficient = 0.83, *p* < 0.001). For example, *GNAQ_B* displayed a high melting temperature for both UMI design X and reference UMI. However, there was no correlation between melting temperature and the GC content of forward target primer sequence (Additional file 1: Fig. 8E).

**Fig. 4:**
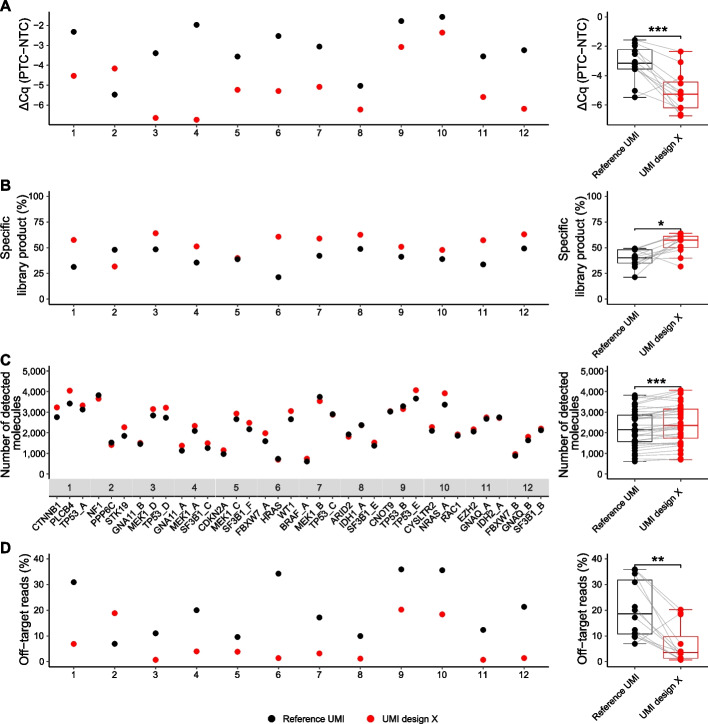
Evaluation of 12 different tri-plexes. Twelve tri-plexes were analyzed with UMI design X and reference UMI using 20 ng DNA. **A** Relative specificity using quantitative PCR. Mean ΔCq was calculated as the difference in cycle of quantification values between samples with DNA (PTC) and no template control (NTC). Mean value for each tri-plex is shown, *n* = 3. Box plots of all mean values are shown to the right. ***p ≤ 0.001, Wilcoxon signed-rank test, *n* = 12. **B** Specificity based on correct library product formation using parallel capillary electrophoresis. The percentage of specific library products relative total DNA amount is shown. Mean value for each tri-plex is shown, *n* = 3. Box plots of mean values are shown to the right. *p ≤ 0.05, Wilcoxon signed-rank test, *n* = 12. **C** Number of detected molecules assessed by digital sequencing. Mean value for each individual assay is shown, *n* = 3. Box plots of all mean values are shown to the right. ***p ≤ 0.001, Wilcoxon signed-rank test, *n* = 36. **D** Fraction of off-target sequence reads. Mean value for each tri-plex is shown, *n* = 3. Box plots of all mean values are shown to the right. **p ≤ 0.01, Wilcoxon signed-rank test, *n* = 12

To test the performance of structured UMI design X in multiplexing, we analyzed 12 different tri-plexes. The tri-plexes were selected to avoid overlapping amplicons but otherwise randomly combined. Figure 4A shows that the relative specificities when assessed by quantitative PCR were improved in 11 of the 12 tri-plexes using UMI design X compared with reference UMI. The tri-plex that performed worse with UMI design X contained the *PPP6C* assay. The mean improvement in relative specificity was 3.9 times. The same tri-plexes also demonstrated improved performance when evaluating specificity in library construction using parallel capillary electrophoresis. The amount of specific library products improved from 40 to 53% (Fig. 4B). In conclusion, our data show that structured UMIs enhance individual assay and panel performance, resulting in improved library purity.

### Structured UMI design improves sequencing performance and the ability to detect target molecules

We sequenced all tri-plexes to test the effects of structured UMIs on coverage. In digital sequencing, coverage can be expressed as the absolute number of detected molecules in samples. Raw sequencing reads were collapsed into consensus reads based on UMI families using a cutoff ≥ 3 reads per UMI family. In our sequencing approach, two consensus reads corresponds to about one original target DNA molecule in the sample [10]. Twenty-seven of 36 individual assays detected higher number of target molecules using UMI design X compared with reference UMI, where the typical assay detected on average 7.4% more molecules (Fig. 4C). We also compared the number of detected molecules of the individual assays in the tri-plexes with their assay performance as single-plexes. Overall, all assays that detected high number of molecules in tri-plexes had also performed well as single-plexes when assessed by both quantitative PCR and parallel capillary electrophoresis (Additional file 1: Fig. S9).

To further assess the effect of improved assay performance, we quantified the fraction of off-target sequencing reads (Fig. 4D). All but one tri-plex generated lower fractions of off-target reads with UMI design X compared with reference UMI. The mean fraction of off-target reads was reduced from 20 to 6.7%. Finally, we sequenced a 16-plex comparing UMI design X and reference UMI (Additional file 1: Fig. S10). Twelve of 16 assays detected more target molecules using UMI design X compared with reference UMI, with a mean improvement of 10%. The number of detected molecules of individual assays in the 16-plex correlated to the performance of single-plexes and tri-plexes (Additional file 1: Fig. S11). The mean fraction of off-target reads was reduced from 46 to 20% using UMI design X (Additional file 1: Fig. S12).

To test the ability to analyze DNA at low concentrations, we sequenced one tri-plex, applying a dilution series of DNA ranging from 27 to 0.33 ng (Additional file 1: Fig. S13). All three assays displayed linearity over the entire range between observed number of detected molecules and loaded DNA amounts.

### Structured UMI design enables ultrasensitive variant allele detection and increases the sensitivity to detect mutated DNA molecules

**Fig. 5:**
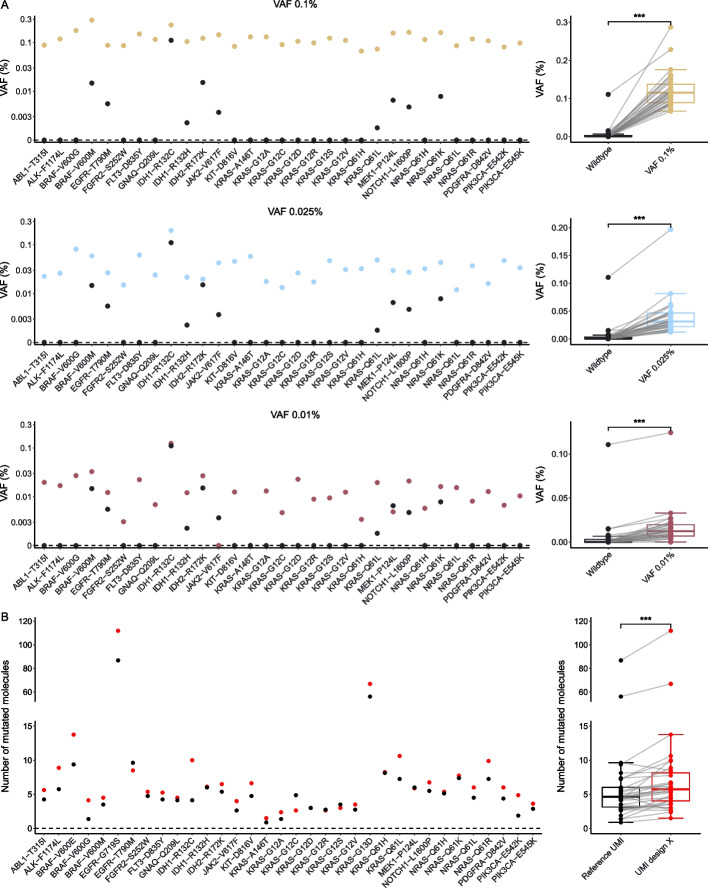
Sensitivity to detect mutated DNA molecules. **A** A hot-spot mutation panel that consists of 20 individual assays was analyzed with three different dilutions of mutations standardized control material using UMI design X. Thirty-one mutations that were possible to dilute in wildtype control material were assessed in 100 ng DNA. The expected variant allele frequency ranged approximately around 0.1%, 0.025%, and 0.01% (Additional file 2: Table S1). The total number of detected molecules for each assay was uniform among all analyzed samples (Additional file 1: Fig. S21). Mean value for each mutation is shown, *n* = 4. Box plots of all mean values are shown to the right. ***p ≤ 0.001, Wilcoxon signed-rank test, *n* = 31. **B** The hot-spot mutation panel was used to assess the difference between UMI design X and reference UMI. The number of mutated molecules was quantified in 10 ng DNA. Note that three mutations were present also in the wildtype material, resulting in a total of 34 mutations. Mean value for each mutation is shown, *n* = 4. Box plots of all mean values are shown to the right. ***p ≤ 0.001, Wilcoxon signed-rank test, *n* = 34

To demonstrate the ability to detect variant allele frequencies < 0.1% with UMI design X, we designed a 20-plex hot-spot mutation panel targeting clinically relevant mutations and analyzed standardized control material with 31 known single nucleotide variants. The amplicon lengths of all assays were short (≤ 110 nucleotides) to enable analysis of fragmented DNA, which is typical in liquid biopsies [15]. Of the 20 individual assays, 17 displayed improved assay performance using quantitative PCR when comparing UMI design X to reference UMI, while all 20 assays showed increased specificity in the library construction based on parallel capillary electrophoresis (Additional file 1: Fig. S14). We analyzed three dilutions of the standardized control material with mean variant allele frequency of 0.1%, 0.025%, and 0.01% (Fig. 5A). We detected all 31 variants for the 0.1% and 0.025% samples and for the 0.01% samples 29 of 31 variants were detectable. For the 0.01% dilution samples, the number of detected mutated molecules were on average between 0 and 2 molecules per mutations, resulting in increased variability (Additional file 2: Tables S1–S2). The mean error rate was < 0.1% for all assays in the hot-spot panel data as well as in the tri-plexes and 16-plex data (Additional file 1: Fig. S15). We conclude that SiMSen-Seq with UMI design X can reliably detect variant allele frequencies < 0.1%.

The use of UMIs facilitates bioinformatical correction of sequencing errors, enabling ultrasensitive mutation detection (Additional file 1: Fig. S16) [6, 10, 16]. As expected, we detected no difference in error rates between UMI design X and reference UMI, since the bioinformatical use of UMI is independent on its structure (Additional file 1: Fig. S15). Instead, we hypothesized that structured UMIs would enhance the capacity to detect mutated DNA molecules due to the improved ability to amplify target DNA sequences. This ability will be useful when the number of mutated molecules is low in limited sample sizes. Hence, we chose to analyze samples at a concentration where we expected about 5 mutated DNA molecules. At this molecule number, the chance of sampling at least 1 molecule is > 99%, thus minimizing confounding effects in downstream data analysis [15]. Here, 34 single nucleotide variants were assessed, three additional compared with the low variant allele frequency test, since mutations in the wildtype samples also were analyzed. Our hot-spot mutation panel detected all 34 existing single nucleotide variants (Fig. 5B and Additional file 2: Tables S1–S2). We detected more mutated DNA molecules for 28 of 34 different mutations using UMI design X compared with reference UMI. We detected on average 34.7% more mutated DNA molecules using UMI design X. The number of detected mutated DNA molecules was above the background noise levels in control material without spiked-in mutations for 32 of 34 specific mutations and the sequencing error rates were at similar levels for both UMI design X and reference UMI (Additional file 1: Fig. S15A and Additional file 2: Tables S1–S2). At most nucleotide positions, we detected zero mutated DNA molecules in the reference material without spiked-in mutations. However, for two mutations (*IDH1* R132C and *IDH2* R172K), we observed similar levels of mutated DNA molecules in all tested DNA samples, indicating that this background is of technical origin but not related to UMI design.

The overall number of detected molecules, including mutated DNA molecules, was also increased in 18 of 20 individual assays, where the typical assay amplified on average 55% more molecules using UMI design X compared with reference UMI (Additional file 1: Fig. S17). The mean improvements when detecting total number of molecules and mutant DNA molecules were somewhat different, since the number of single nucleotide variants was not equal among the individual assays. The distributions of UMI-family sizes were almost identical when comparing UMI design X with reference UMI data (Additional file 1: Fig. S18), indicating that the library amplification of different UMIs was not affected between these two designs. In conclusion, our data show that the use of structured UMIs increases the sensitivity to detect mutated target DNA molecules due to improved assay performance that enhances amplification of target molecules.

### Personalized ctDNA panels using structured UMIs enable detection of < 0.01% variant allele frequencies in clinical samples

Mutation detection in liquid biopsies is becoming an important tool in cancer management, such as in diagnosis, treatment prediction, prognostication, monitoring of treatment efficacy, and early detection of treatment resistance as well as relapse [9, 17]. A challenge is that the amount of cell-free DNA is typically sparse, often below 10 ng per mL blood plasma for healthy individuals [18, 19], where 1 ng DNA corresponds to approximately 310 target molecules for single locus sequences [20]. Hence, the sensitivity to detect mutated molecules is determined by both the amount of cell-free DNA and the applied method. To improve the clinical ctDNA sensitivity, the number of simultaneously assessed mutations needs to be increased.

**Fig. 6:**
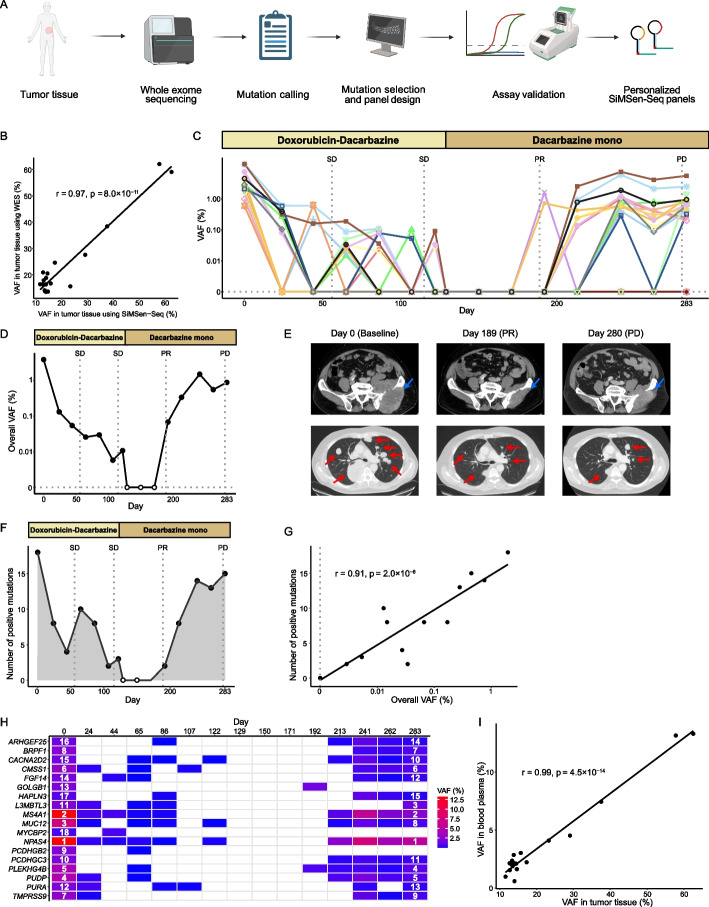
Circulating tumor-DNA analysis in leiomyosarcoma. **A** Schematic workflow for identification and design of personalized ctDNA panels using SiMSen-Seq with structured UMIs (partially created in BioRender. Andersson, D. (2024) https://BioRender.com/z62n478). **B** Validation of mutations identified in tumor tissue using whole exome sequencing with a personalized SiMSen-Seq ctDNA panel. The Pearson correlation coefficient (*r*) was calculated, *n* = 18. VAF, variant allele frequency; WES, whole exome sequencing. **C** Variant allele frequencies for 18 patient-specific mutations in blood plasma during palliative chemotherapy in a patient diagnosed with leiomyosarcoma. Plasma samples were analyzed with a personalized SiMSen-Seq ctDNA panel. Treatments and results of radiological evaluations are shown at the top of the diagram. The variant allele frequencies are shown in log_10_-scale. The corresponding diagram with number of ctDNA molecules per mL plasma is shown in Additional file 1: Fig. S20B. PR, partial response; SD, stable disease; PD, progressive disease. **D** Overall variant allele frequency in blood plasma. The overall variant allele frequency shown in log_10_-scale was calculated as the total number of all detected ctDNA molecules divided by the total number of detected molecules for all assays. Treatments and results of radiological evaluations are shown at the top of the diagram. Open circles represent no detectable ctDNA. The corresponding diagram with number of ctDNA molecules per mL plasma is shown in Additional file 1: Fig. S20C. **E** Computed tomography of the pelvis (top) and the chest (bottom) at days 0, 189, and 280. Blue arrows indicate the primary tumor in the left ilium. Red arrows indicate lung metastases. **F** Number of detected mutations over time. Open circles represent no detectable ctDNA. **G** Number of detected mutations versus overall variant allele frequency. The overall variant allele frequencies are shown in log_10_-scale. The Pearson correlation coefficient (*r*) was calculated, *n* = 18. **H** Mutation heterogeneity over time. The heat map shows the variant allele frequency over time for each individual mutation. The rankings of mutations based on variant allele frequencies for the first and last time points are shown. Time points with no detectable ctDNA are shown in white color. **I** Variant allele frequencies in blood plasma versus tumor tissue. The Pearson correlation coefficient (*r*) was calculated, *n* = 18

An emerging strategy is to apply patient-specific ctDNA panels targeting multiple mutations, initially identified in tumor tissue through whole exome sequencing (Fig. 6A). Here, we explored this concept in a patient diagnosed with leiomyosarcoma. First, we identified tumor-specific mutations using whole exome sequencing. Then, we designed a personalized ctDNA panel using UMI design X targeting 18 of the single nucleotide variants with the highest variant allele frequencies. Additional file 1: Fig. S19 outlines a step-by-step guideline to design and evaluate assays and panels. To verify all selected mutations and to validate the personalized ctDNA panel, we re-sequenced the tumor tissue DNA with SiMSen-Seq (Fig. 6B). We also analyzed reference DNA extracted from patient blood cells to confirm that all mutations in the whole exome data were somatic (Additional file 1: Fig. S20A). The error rate of the personalized ctDNA panel was 0.0011% for the selected mutation panel. Fifteen blood plasma samples were collected over a period of 283 days, each sampled prior to a new cycle of chemotherapy. Figure 6C–D and Additional file: Fig. S20B–C show the dynamics of ctDNA levels in relation to treatment and clinical routine assessment. We quantified both the variant allele frequencies of individual mutations as well as the overall variant allele frequency. At the first time point, the frequencies of the individual mutations ranged between 0.6 and 13.3% and the overall variant allele frequency was 3.9%. The ctDNA level decreased until day 107, where the overall variant allele frequency was 0.0058% with only two individual mutations detected. At days 129, 150, and 171, no ctDNA was detected and the patient showed partial response using radiological assessment at day 189 (Fig. 6E). At day 192, ctDNA was again detected with an overall variant allele frequency of 0.068%. The overall variant allele frequency continued to increase until day 241 and then remained at a similar level during the last two time points (days 262 and 283). At day 280, a radiologically progressive disease was confirmed (Fig. 6E). The number of individually detected mutations varied over time, where the overall variant allele frequency correlated with the total number of detected mutations demonstrating the importance of assessing multiple mutations to gain high sensitivity detecting ctDNA (Fig. 6F–G). The mutations that were detected for the three plasma samples with lowest overall variant allele frequencies (days 107, 122, and 192) were also different from each other, illustrating the relevance of using personalized ctDNA panels. To assess the dynamic of different mutations in released ctDNA, we ranked the individual mutations based on variant allele frequency and compared them over time (Fig. 6H). We observed some variations in allele frequencies over time but no major differences between the individual mutations, illustrated by the fact that 16 out of 18 mutations remained detectable at the last time point even if the overall variant allele frequency was > 4 times lower compared with the first time point. We also observed a correlation between the variant allele frequency observed in the first blood plasma sample and the tumor tissue (Fig. 6I). In summary, personalized ctDNA panels using digital sequencing with structured UMIs enable detection of variant allele frequencies < < 0.1% in blood plasma.

## Discussion

Digital sequencing approaches using UMIs are emerging tools in numerous basic research and clinical applications, such as cancer screening [16], immune repertoire profiling [21], organ transplantation [22], short tandem repeats analysis in forensics [23, 24], RNA editing [25], and prenatal testing [26]. For UMI-based correction of polymerase-induced errors to work, each original molecule labeled with a specific UMI must be detected multiple times. Consequently, digital sequencing needs ultradeep sequencing and is therefore often limited to targeted DNA analysis [9]. The challenge of using UMIs in library construction is the massive formation of non-specific PCR products. In this study, we have addressed this issue by using structured UMIs that reduce the number of unwanted primer interactions. Our data show that by inserting predefined nucleotides at specific positions in the randomized UMI sequence it is possible to significantly improve assay performance in digital sequencing, including specificity, library yield, on-target reads, and sensitivity to detect target DNA molecules compared with conventional unstructured UMIs. Overall improved assay performance also reduces assay development time and enables use of limited starting material.

We hypothesized that the use of structured UMIs could be efficient in digital sequencing, particularly in PCR-based library construction approaches, such as SiMSen-Seq [10], Safe-SeqS [6], TARDIS [27], and single-molecule molecular inversion probes [8]. Here, we used SiMSen-Seq that is characterized by a flexible workflow that allows for simple assay design, optimization, and validation of small- to medium-sized panels [14]. The stem structure in the SiMSen-Seq barcoding primer (Fig. 1B) improves assay performance by reducing the formation of non-specific PCR products during the annealing step of barcoding PCR [10, 28, 29]. This is not purely a result from minimizing the non-specific interactions of the UMI to other sequences but also unintended interactions of the adapter sequence. An intrinsic disadvantage of the SiMSen-Seq primer design is that the primer, including the open loop structure, may still interact with other sequences, including kissing-loop interactions [30, 31]. This motivates improvements, such as introduction of structured UMIs. In our study, all structured UMIs displayed improved overall assay performance for all assessed parameters compared with reference UMI. We observed that related UMI designs with lower UMI diversity generally performed better than related designs with higher diversity, which was expected since higher UMI diversity intrinsically increases the risk of interactions to any other DNA sequence in the reaction. In contrast, the number of structured elements in the UMI design was of less importance. For example, UMI designs XVII and XVIII that contain structured components in every second nucleotide position were not superior the other designs. The additional stem sequence of UMI design XIX only showed minor improvement compared to unstructured reference UMI, indicating that the original stem is sufficient to protect the UMI structure. This is also supported by the fact that lower UMI diversity, as seen in UMI design II, or a design that may form more UMI-UMI interactions, as exemplified by UMI designs VI and VII, performed similarly to the other well-performing UMI designs. We speculate that structured UMIs have less interactions with non-UMI sequences during library construction and that the polymerization reaction performs better. We argue that UMI design X is a relevant choice in SiMSen-Seq as it maintains high UMI diversity and performed well in all tested metrics in relation to the other UMI designs. This is further supported by the data demonstrating that UMI design X systematically performed well in all downstream validation analysis compared with unstructured reference UMI. However, we expect that different digital sequencing protocols beyond SiMSen-Seq may be affected somewhat differently by various structured UMI designs, where some digital sequencing approaches may even benefit more since they do not have the partly protective stem-loop structure.

We demonstrated that variant allele frequencies < 0.1% were reliably detected in standardized control material and clinical samples. The theoretical sensitivity of most digital sequencing strategies that utilize UMIs is similar when correcting for polymerase-induced errors. Our data showed no difference in the degree of correction of polymerase-induced errors using structured UMIs compared with reference UMI. Bioinformatically, it has been demonstrated that UMI designs with a balanced GC content and without long repetitive sequences perform optimally by minimizing errors in the actual UMI during sequencing, which otherwise may cause biases [32]. Some of our UMI designs utilized this concept, especially UMI designs II–VII. However, in SiMSen-Seq this issue is expected to be essentially neglectable since relatively short UMI designs are used and because all DNA molecules are targeted multiple times with several different UMIs.

Interestingly, we observed an increased ability to detect target DNA molecules using structured UMIs that resulted in improved sensitivity to find mutant DNA molecules. We observed on average 35% more mutant molecules per mutation. In applications with limited amounts of DNA, such as liquid biopsies, this increased sensitivity is highly relevant since all available cell-free DNA is typically analyzed. In contrast, in applications where the amount of starting material is not limited this improvement will have minor effect. The underlying reason for the experimentally improved sensitivity using structured UMIs is not known, but one explanation may be that barcoding primers with structured UMIs are more effective in the initial labeling and amplification of target molecules since these primers are less likely to interact with each other, an effect that cannot be compensated by increased primer concentrations [33]. A small number of assays performed somewhat worse using structured UMIs. One cause of poorly performing assays is errors that occur during chemical synthesis of oligonucleotides, which may result in abnormal formation of non-specific PCR products during library constructions [34]. Potential disadvantages of using structured UMIs in digital sequencing are that the primers used for library construction are a few nucleotides longer to synthesize and that sequencing requires additional cycles to compensate for UMI length. However, in most applications these limitations will be insignificant.

We analyzed longitudinally collected blood plasma samples from a patient being treated for metastatic leiomyosarcoma. The gold standard of sarcoma treatment relies on radiological evaluations with no reliable blood-based biomarkers available. Other studies have only explored shallow whole-genome sequencing and targeted deep sequencing for ctDNA as response marker in leiomyosarcoma [35–38]. These approaches suffer from low sensitivity and the risk of detecting false positives. Here, we have shown that treatment monitoring using a personalized ctDNA panel is a feasible and relevant option, where the ctDNA levels closely correlated with clinical outcomes. Interestingly, the patient was ctDNA positive already 3 days after a partial response was radiologically observed. A progressive disease was then confirmed 3 months later. Our data using personalized ctDNA panels displayed potential clinical utility, but analysis of a larger patient cohort is needed to determine significance.

## Conclusions

We demonstrate that the use of structured UMIs provides several experimental benefits in digital sequencing using SiMSen-Seq, without any obvious drawbacks. Data show that variant allele frequencies < 0.1% can be reliably detected in standardized control material and clinical samples, although further studies are needed to determine if all structured UMI designs display similar properties in specific sequencing approaches or if some are more advantageous than others.

## Methods

### Clinical samples and DNA extractions

The patient was included in the SARKOMTEST-study. Clinical data, including treatment regimen and radiological response assessment, were collected during the study. Radiological response assessments were evaluated according to RECIST version 1.1 criteria.

Blood samples were collected in K_2_EDTA tubes (#456,243, Hettich Labinstrument) and cf-DNA/cf-RNA Preservative Tubes (#63,960, Norgen Biotek). Plasma was isolated from K_2_EDTA tubes by centrifugation at 2000 *g* for 10 min at room temperature and from cf-DNA/cf-RNA Preservative Tubes by centrifugation at 425 *g* for 20 min. Plasma, buffy coat, and cell fraction were stored at − 80 °C.

Tumor tissue DNA was extracted from formalin-fixed paraffin-embedded material using GeneRead FFPE DNA kit (#180,134, Qiagen), while DNA from buffy coat was isolated using QIAamp DNA Blood Mini Kit (#51,104, Qiagen). Circulating cell-free DNA was extracted from blood plasma collected in K_2_EDTA tubes with the QIAsymphony system using QIAsymphony DSP Circulating DNA Kit (#937,556, Qiagen) following a second centrifugation at 16,000 *g* for 10 min at room temperature, and from blood plasma collected in cf-DNA/cf-RNA Preservative Tubes using QIAamp Circulating Nucleic Acid Kit (#55,114, Qiagen). All DNA isolation protocols were performed according to the manufacturer’s instructions. The concentration of DNA was quantified with Qubit 3.0 Fluorometer (Thermo Fisher Scientific).

### Library construction

Detailed SiMSen-Seq protocol is previously described [14]. Target primers for the hot-spot mutation panel were designed using PanelPlex (DNA Software). The SiMSen-Seq protocol consists of two rounds of PCR followed by library purification and sequencing. The first 10 μL barcoding PCR contained 0.05 U Platinum SuperFi DNA polymerase, 1 × SuperFi Buffer (both #12,351,010, Thermo Fisher Scientific), 0.2 mM deoxyribonucleotide triphosphate (#D7295, Merck), 40 nM of each primer (Ultramer, Integrated DNA Technologies, Additional file 3: Tables S3–S4), 0.5 M L-carnitine inner salt (#C0158, Sigma-Aldrich), and 10–20 ng human genomic DNA (#11,691,112,001, Roche Diagnostics). Standard curves were generated with 27, 10, 3, 1, and 0.33 ng DNA per reaction. For low variant allele frequency tests, 10–100 ng True-Q 7 (#HD734, Horizon) or True-Q 0 (#HD752, Horizon) standardized control were used. The True-Q 7 with mean variant allele frequency of 1.3% was diluted in True-Q 0 to generate 0.1%, 0.025%, and 0.01% controls. The following thermal program was used on a T100 Thermal Cycler (Bio-Rad Laboratories): 30 s at 98 °C, 3 cycles of amplification (98 °C for 10 s, 62 °C for 6 min, 72 °C for 30 s), 15 min at 65 °C, and 15 min at 95 °C. The DNA polymerase was inactivated by adding 20 µL TE-buffer (pH 8.0, #AM9858, Thermo Fisher Scientific) supplemented with 30–45 ng/μL *Streptomyces griseus* protease (#P5147, Merck) to the reaction at the start of the 65 °C incubation step. The second adapter PCR was performed in 40 μL reactions, containing 1 × Q5 Hot Start High-Fidelity Master Mix (#M0494, New England Biolabs), 400 nM of each Illumina adapter primer (desalted, Merck, Additional file 3: Table S5), and 10 μL diluted barcoding PCR product. The following thermal program was used on a T100 Thermal Cycler: 98 °C for 3 min, followed by 26–30 cycles of amplification (98 °C for 10 s, 80 °C for 1 s, 72 °C for 30 s, 76 °C for 30 s, the ramping rate was 0.2 °C/s between 80 °C, 72 °C, and 76 °C). The 16-plex was experimentally performed as an 18-plex reaction, but two assays (*TP53_C* and *TP53_D*) were omitted in downstream data analysis due to overlapping amplicons. Library construction for clinical samples was performed in 15 μL barcoding PCR using 0.1 U Platinum SuperFi polymerase II and 1 × SuperFi II Buffer (both #12,361,010, Thermo Fisher Scientific). The thermal program was 3 min at 98 °C, 3 cycles of amplification (98 °C for 10 s, 60 °C for 6 min, 72 °C for 30 s), 15 min at 65 °C, and 15 min at 95 °C. The adapter PCR was performed in 60 μL with 1 × Platinum SuperFi II PCR Master Mix (#12,368,010, Thermo Fisher Scientific) and 15 μL diluted barcoding PCR product.

### Quantitative PCR

The amount of UMI-labeled DNA was quantified using quantitative PCR and a CFX384 Touch Real-Time PCR Detection System (Bio-Rad Laboratories). Two microliters diluted PCR product from the first barcoding PCR reaction was analyzed in 10 µL reactions, containing 1 × TATAA SYBR GrandMaster Mix Low Rox (#TA01-3750LR, TATAA Biocenter) and 400 nM of each Illumina adapter primer (Additional file 3: Table S5). The following thermal program was used: 98 °C for 3 min, 45 cycles of amplification (98 °C for 10 s, 80 °C for 1 s, 72 °C for 30 s, 76 °C for 30 s, all with ramping rate at 0.2 °C/s), and melting curve analysis ranging from 60 to 95 °C, 0.5 °C/s increments. Cycle of quantification values were determined by regression using the Bio-Rad CFX Maestro software (version 4.1, Bio-Rad Laboratories).

### Parallel capillary electrophoresis

The PCR products from the second adapter PCR were evaluated on a 5200 Fragment Analyzer using either dsDNA 915 Reagent kit (35–5000 base pairs, #DNF-915-K1000, Agilent Technologies) or HS NGS Fragment kit (1–6000 base pairs, #DNF-474–0500, Agilent Technologies), according to the manufacturer’s instructions. Library purity was calculated as the fraction of specific library product (210–300 base pairs) compared with total DNA amount. Analysis was performed using ProSize Data Analysis Software (version 4.0.0.3, Agilent Technologies).

### Melting curve analysis

The melting temperature of the stem-loop structure in barcoding primers was determined by high resolution melting curve analysis using a Mic PCR machine (Bio Molecular Systems). The 20 µL reaction contained 1 × SuperFi Buffer, 1 × SYBR Green I nucleic acid gel stain (#S9430, Sigma-Aldrich), and 1 µM forward barcoding primer (Additional file 3: Tables S3–S4). The temperature profile was 95 °C for 3 min, 60 °C for 3 min, followed by melting analysis from 60 to 90 °C with ramping at 0.03 °C/s. Melting curve analysis was performed using the Mic quantitative PCR software (version 2.10.03, Bio Molecular Systems).

### Sequencing

Library concentrations were assessed on a 5200 Fragment Analyzer (Agilent Technologies) using the DNF-474 HS NGS Fragment Kit (1–6000 base pairs, #DNF-474–1000, Agilent Technologies). Samples were pooled to enable similar sequencing depth across all samples. The pooled library was purified with a Pippin Prep (Sage Science) using a 2% agarose, dye-free, Pippin Gel Cassette (100–600 base pairs, #CDF2010, Sage Science), according to manufacturer’s instructions. Purified libraries were quantified using quantitative PCR. Briefly, 2 µL of the purified libraries was quantified in 10 µL reactions, containing 1 × TATAA SYBR GrandMaster Mix Low Rox (#TA01-3750LR, TATAA Biocenter) and 400 nM of each Illumina adapter primer (5′-AATGATACGGCGACCACCGA-3′ and 5′-CAAGCAGAAGACGGCATACGA-3′). The concentration was estimated using a standard curve with adjustment to the average library size. Sequencing was performed on either a MiniSeq (Illumina) with 20% PhiX (#FC-110–3001, Illumina) using the MiniSeq High Output Reagent Kit (#FC-420–1002, Illumina) or NextSeq 550Dx (Illumina) with 20% PhiX using NextSeq 500/550 Output Kit v2.5 (#20,024,904, Illumina). The final library concentrations were 1.0–1.8 pM and sequencing was performed in single-end and 150 base pairs mode. All samples were sequenced to a sample-specific depth to provide the required coverage to enable UMI-error correction [39]. Raw sequencing data for all samples are available at the National Center for Biotechnology Information Sequence Read Archive (PRJNA844028) [40].

Whole exome sequencing was performed by the core facility, SNP&SEQ Technology Platform (Uppsala, Sweden).

### Data analysis

Digital sequencing data was analyzed using the UMIErrorCorrect pipeline [39]. The pipeline aligned reads to the hg38 reference genome using the Burrows-Wheeler aligner [41], clustering reads into UMI families based on their UMI sequence and chromosomal position. Each UMI family was subsequently collapsed to one consensus read, counting the most common nucleotide type at each position. Here, final data were filtered using a UMI family size cutoff ≥ 3. The number of off-target reads was calculated as the difference between the total number of observed reads and the number of reads aligned to intended target sequences. The error rate was calculated as the total number of non-reference alleles per nucleotide position divided by coverage. Single nucleotide polymorphism positions identified in the Single Nucleotide Polymorphism Database were excluded [42]. Single nucleotide mutations in the standardized control material were identified with UMIErrorCorrect. Insertions and deletions were omitted from analysis since UMIErrorCorrect is not optimized for these types of mutations.

Interactions between UMIs were simulated using a custom Python script. First, 10,000 different UMIs with a given UMI structure were generated. Subsequently, each UMI was compared to the sequence of all other UMIs in a pairwise manner. Two sequences were considered to form UMI-UMI interactions if they base paired in at least six consecutive nucleotide positions and where at least three pairs contained G-C base pairing. In the simulation tool, it is possible to vary number of UMIs, UMI length, UMI structure, and number of overlapping base pairs including GC base pairs.

The Sarek pipeline was used to call somatic mutations using whole exome sequencing data [43]. Reads were aligned to the hg19 reference genome and identified single-nucleotide variants using Mutect2 [44], Strelka2 [45], and Freebayes [46]. High-confidence variants identified by all three callers were used for downstream analysis.

Statistical tests were performed and figures were generated in R version 4.2.2 [47], using packages ggplot2 [48] and ggpubr [49].

## Supplementary Information

Additional file 1. This file includes multiple supplementary figures ranging from Fig. S1 to S21 [54].

Additional file 2. This file includes supplementary tables raging from Table S1 to S2.

Additional file 3. This file includes supplementary tables raging from Table S3 to S5.

Additional file 4. This file includes supplementary tables raging from Table S6 to S10 with source data for figures.

Additional file 5. This file includes supplementary tables raging from Table S11 to S29 with source data for supplementary figures 3 to 21.

Additional file 6. Peer review history.

## Data Availability

Raw sequencing data have been deposited in the National Center for Biotechnology Information, Sequence Read Archive (PRJNA844028) [40]. The source data for all figures are shown in Additional file 4: Tables S6–S10 and for the supplementary figures in Additional file 5: Tables S11–S29. The UMIErrorCorrect bioinformatics is available at Github https://github.com/stahlberggroup/umierrorcorrect and figshare [50] under the MIT license. The code to simulate UMI-UMI interactions is available at Github https://github.com/stahlberggroup/structuredUMI and figshare [51] under the MIT license. The code to determine off-target sequencing reads is available at Github https://github.com/mlunas/off_target and figshare [52] under the MIT license.
