# Supplementary material for: Digital sequencing is improved by using structured unique molecular identifiers
Source: Genome Biol. 2025 Feb 25;26:37. doi: 10.1186/s13059-025-03504-x (PMC11853513; doi:10.1186/s13059-025-03504-x)
Supplement: Supplementary file 1 — Additional file 1. This file includes multiple supplementary figures ranging from Fig. S1 to S21 [54]. [file 13059_2025_3504_MOESM1_ESM.docx]

# Additional file 1

# Fig. S1

# Fig. S2

# Fig. S3

# Fig. S4

# Fig. S5

# Fig. S6

Fig. S7

Fig. S8

Fig. S9

Fig. S10

Fig. S11

Fig. S12

Fig. S13

Fig. S14

Fig. S15

Fig. S16

Fig. S17

Fig. S18

Fig. S19

Fig. S20

Fig. S21


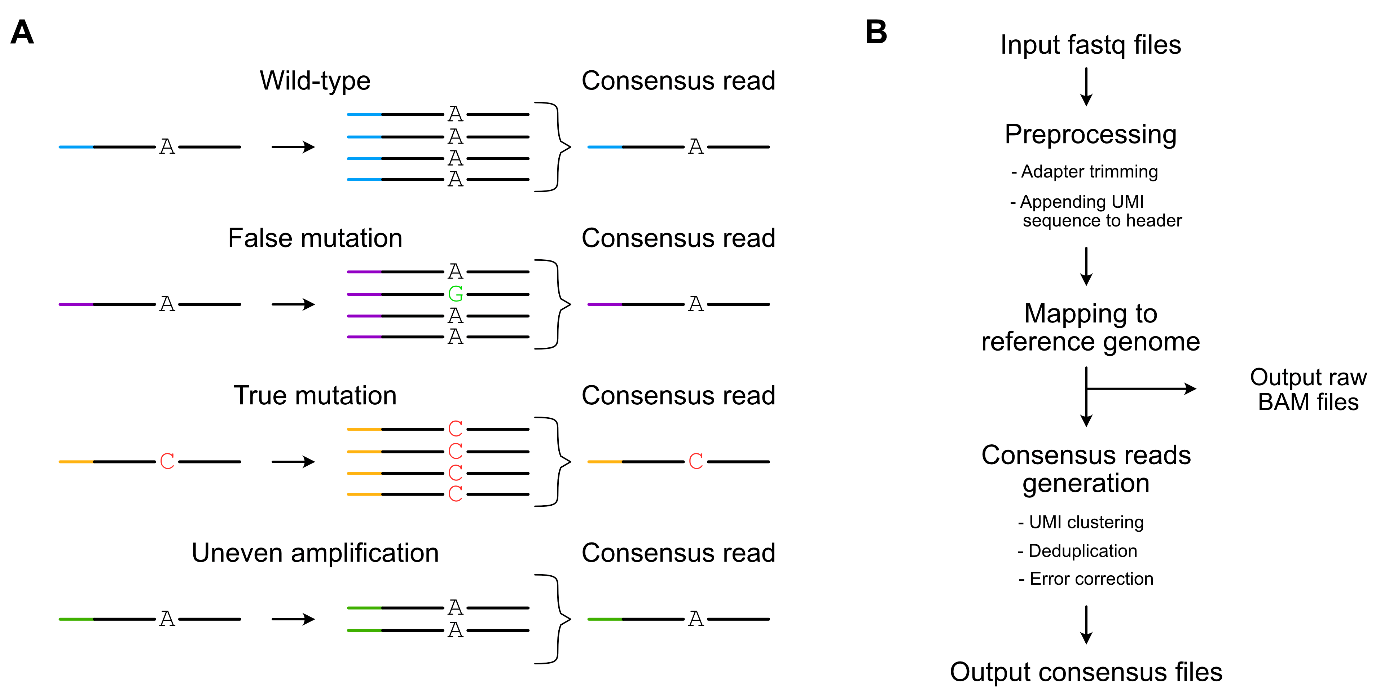


**Fig. S1.** The concept of UMIs in digital sequencing. **A** Each template DNA molecules is labeled with a specific UMI. Polymerase-induced errors can be corrected bioinformatically during consensus read generations, enabling to separate true variants from false variants. The use of UMIs can also minimize quantification biases. **B** Schematic overview of bioinformatics used to analyze UMI-based data. UMIErrorCorrect, a Phyton-based package, was used [39].


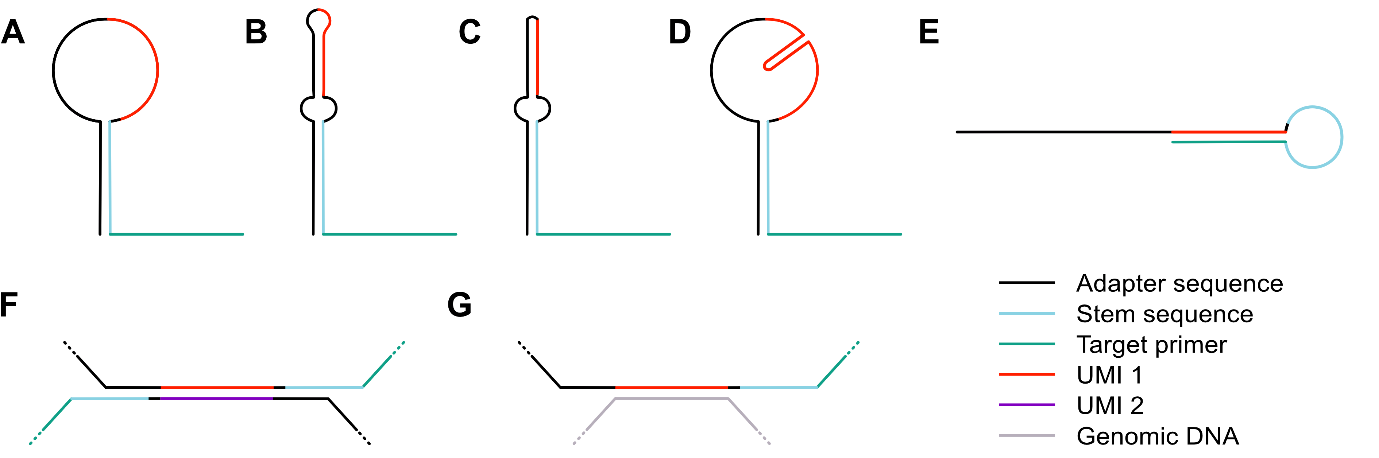


**Fig. S2.** Schematic overview of possible and unintended interactions of forward barcoding primer interactions. **A** Standard SiMSen-Seq forward barcoding primer. **B** Double-stem structure, including UMI design XIX. **C** Double-stem structure where the entire UMI interacts with the adapter sequence. **D** Stem structure within the UMI. **E** Interaction between the UMI and target primer sequence. **F** Dimer formation of two primers with reverse complementary UMIs. **G** Interaction between the UMI and input DNA.


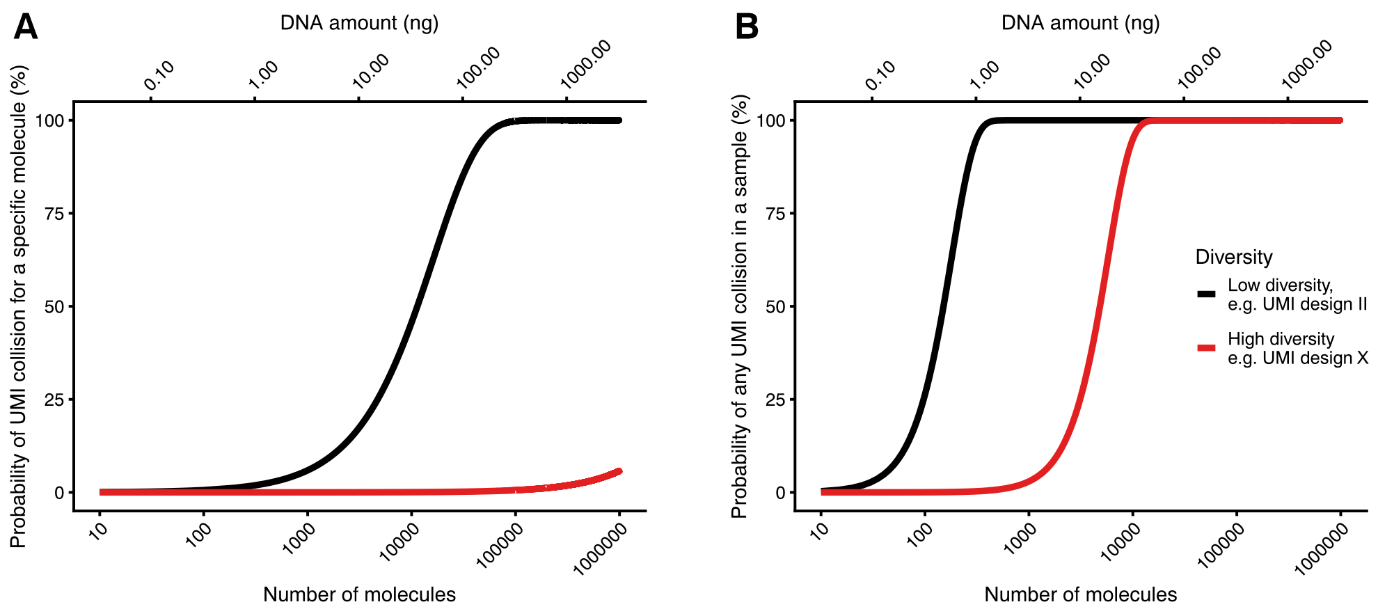


**Fig. S3.** UMI diversity and probability of collisions. **A** The probability that a specific DNA molecule will be labeled by a UMI identical to that of another DNA molecule is shown in relation to UMI diversity and input number of molecules. One nanogram of human DNA corresponds to about 310 haploid genomes [20]. The probability of a specific UMI collision is calculated as $1-\left( \frac{n-1}{n} \right)^{k}$, where *n* is the diversity and *k* is the number of molecules. UMI designs VI (1.68×10^7^) and II (1.64×10^4^) represent the highest and lowest UMI diversities that were experimentally evaluated. **B** The probability that any two or more DNA molecules in the sample will be labeled with identical UMI as a function of UMI diversity and number of molecules. The probability of any UMI collision is calculated as $1-e^{\frac{{-k}^{2}}{2n}}$, where *n* is the UMI diversity and *k* is the number of molecules [54]. Note that the risk of any UMI collision is relatively high. However, for digital sequencing applications aiming at detecting low frequent allele frequencies, the scenario in subplot A is the more relevant case.


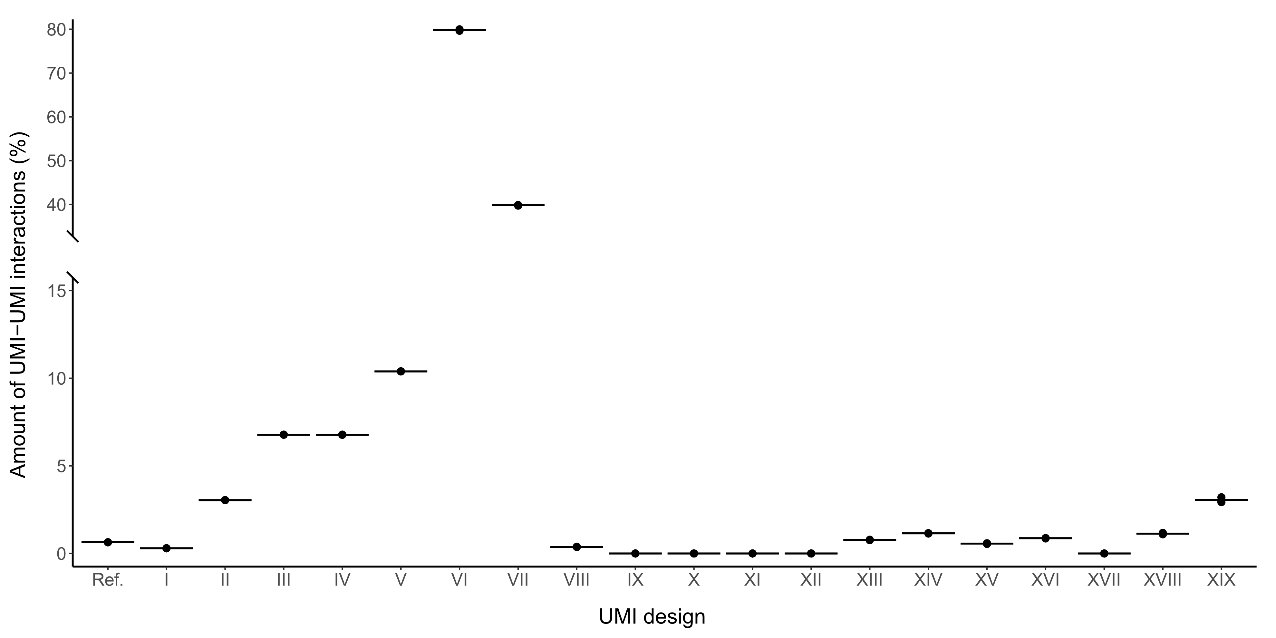
**Fig. S4.** Simulation of UMI-UMI interactions. The number of UMIs that may interact with other UMIs using simulation with 10,000 UMIs. Here, two UMIs were considered to form UMI-UMI interactions if they base paired as hetero-dimers in at least six consecutive nucleotide positions and at least three base pairs contained G-C base pairing. UMI designs IX-XII and XVII did not form any UMI-UMI interactions. The mean is indicated by a bar, n = 3.


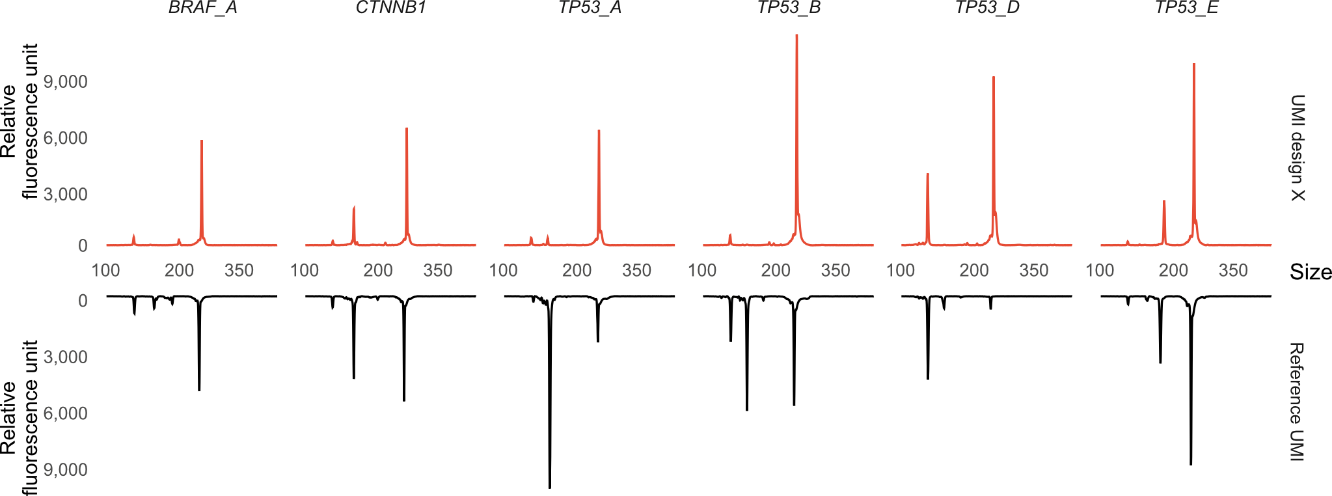


**Fig. S5.** Library yield determined with parallel capillary electrophoresis. Representative electropherograms for UMI design X and reference UMI are shown for six assays, n = 1. Specific PCR products are within the 210 – 300 base pairs range.

**
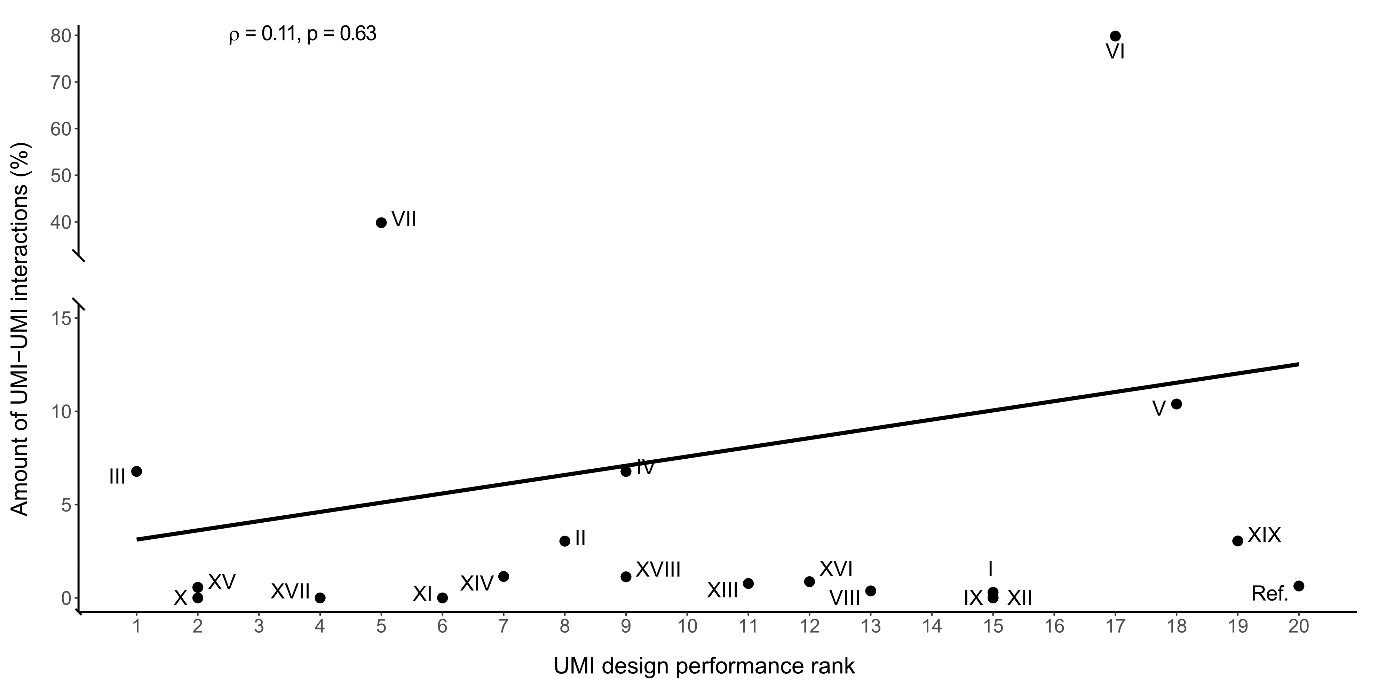
Fig. S6.** Simulation of UMI-UMI interactions in relation to UMI design performance. The amount of UMI-UMI interactions versus UMI design performance rank from Fig. 2E. Spearman’s correlation coefficient (ρ) is calculated. The linear fit is to guide the eye. Ref., reference UMI.


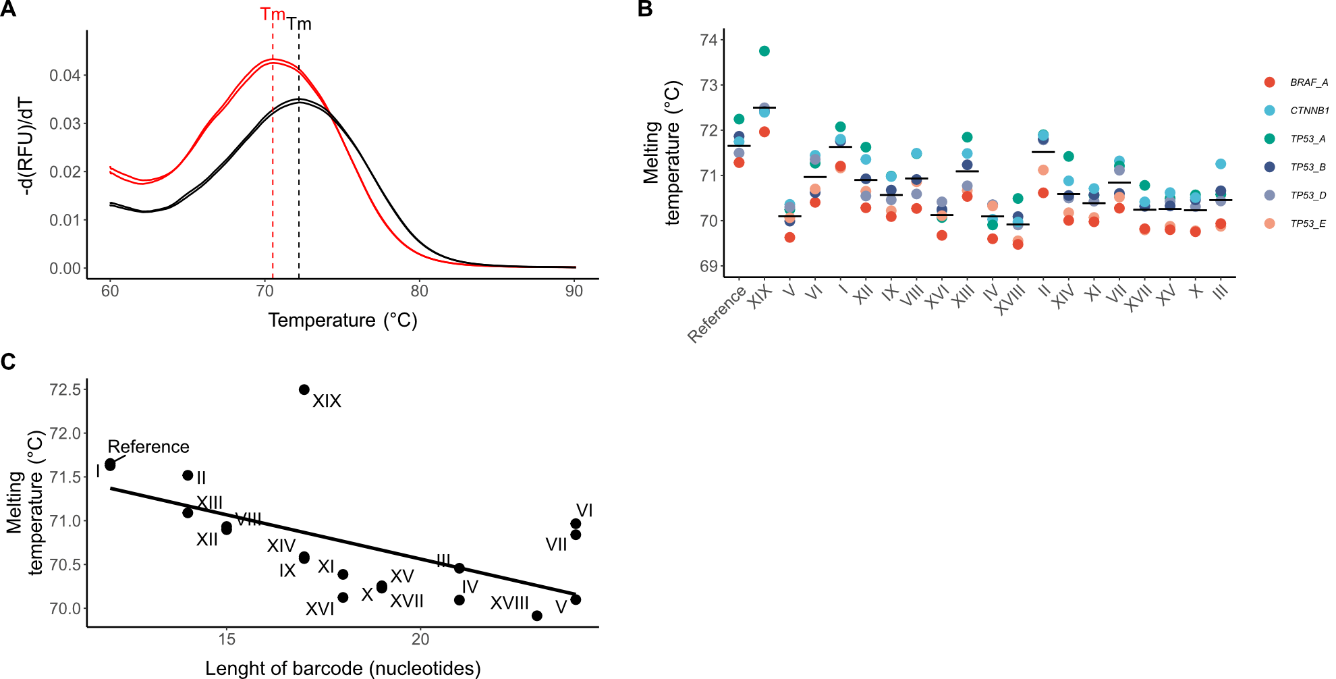


**Fig. S7.** Melting curve analysis. **A** Melting curves are shown for SiMSen-Seq forward primers with UMI design X and reference UMI in red and black, respectively, for assay *TP53_A*. The dotted vertical lines indicate melting temperatures (Tm) of respective UMI design, n = 2. **B** Melting temperatures of the stem structures testing all structured UMI designs and reference UMI. Mean melting temperature is shown for each individual assay, n = 2. Bar indicates mean melting temperature for each UMI design. **C** Mean melting temperature versus length of each UMI design. The Spearman’s correlation coefficient (ρ) was - 0.67, p < 0.01. The linear fit is to guide the eye. RFU; relative fluorescence unit.


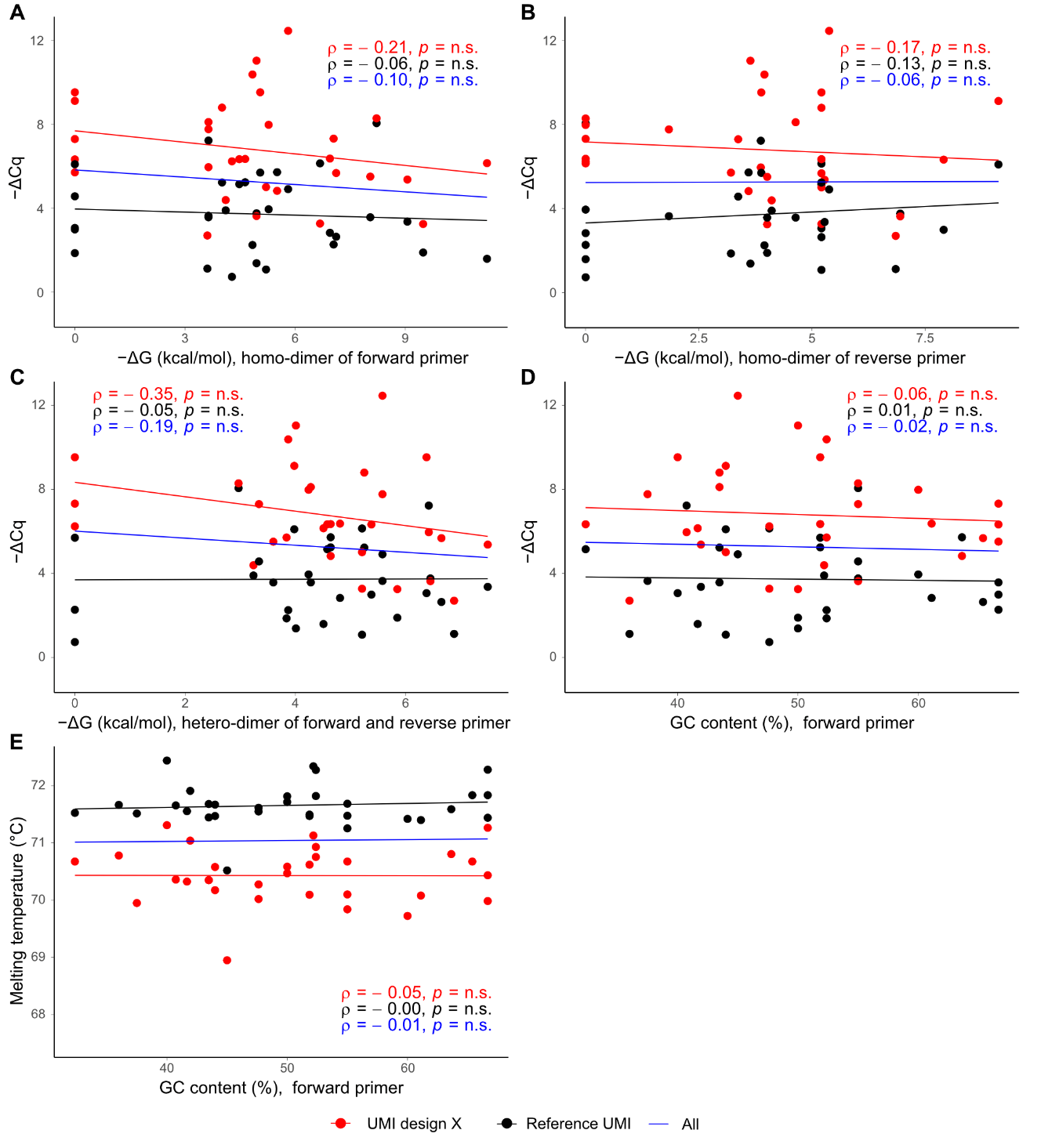


**Fig. S8.** Assay performance in relation to target primer properties. **A** ΔCq versus ΔG of forward target primer’s capacity to form homo-dimer. ΔCq is calculated as the difference in cycle of quantification value for samples using 20 ng DNA and no template control using single-plex data from Figure 3A. Gibbs free energy (ΔG) is estimated as kcal/mol using NetPrimer (https://www.premierbiosoft.com/netprimer/) with default settings at 60 °C. **B** ΔCq versus ΔG of reverse primer’s capacity to form homo-dimer. **C** ΔCq versus ΔG of forward and reverse target primers’ capacity to form hetero-dimer. **D** ΔCq versus the GC content in forward target primer. **E** Melting temperature versus the GC content in forward target primer. Note that it is only the target sequence of each primer that is assessed (Fig. 1C). Spearman’s correlation coefficients (ρ) are calculated for UMI design X and reference UMI separately and all together in all subfigures. Each dot corresponds to one individual assay. The linear fits are to guide the eye. n.s., not significant.

**
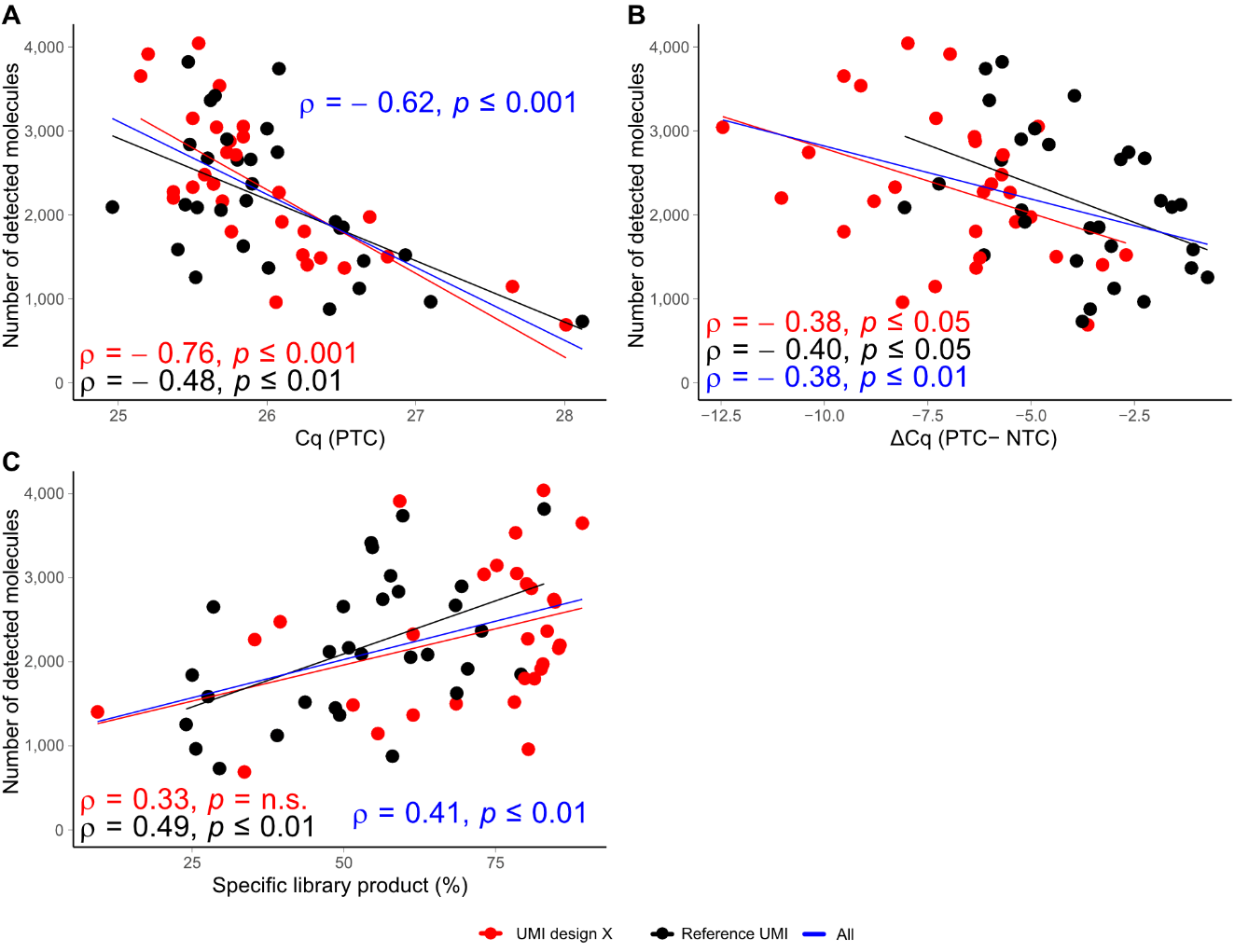
Fig. S9.** Number of detected molecules in 12 tri-plexes in relation to assay performance. **A** The number of detected molecules versus quantitative PCR performance. Number of detected molecules was estimated in the tri-plex data set. Cq is the cycle of quantification value for samples using 20 ng DNA (PTC) determined in the single-plex data set. One cycle corresponds to a two-fold difference in sensitivity to detect target DNA, assuming 100% PCR efficiency and equal fluorescence generation during amplification for all assays. **B** The number of detected molecules versus quantitative PCR performance assessed as ΔCq. Number of detected molecules was estimated in the tri-plex data. ΔCq was calculated as the difference in cycle of quantification values between samples using 20 ng DNA (PTC) and no template control (NTC) using the single-plex data set. ΔCq equals one corresponds to a two-fold difference in assay specificity, assuming 100% PCR efficiency. **C** The number of detected molecules versus library purity using parallel capillary electrophoresis. Number of detected molecules was estimated in the tri-plex data, while library purity data was assessed in the single-plex data set. Spearman’s correlation coefficients (ρ) are calculated for UMI design X and reference UMI separately and all together in all subfigures. Each dot corresponds to one individual assay. The linear fits are to guide the eye. n.s., not significant.


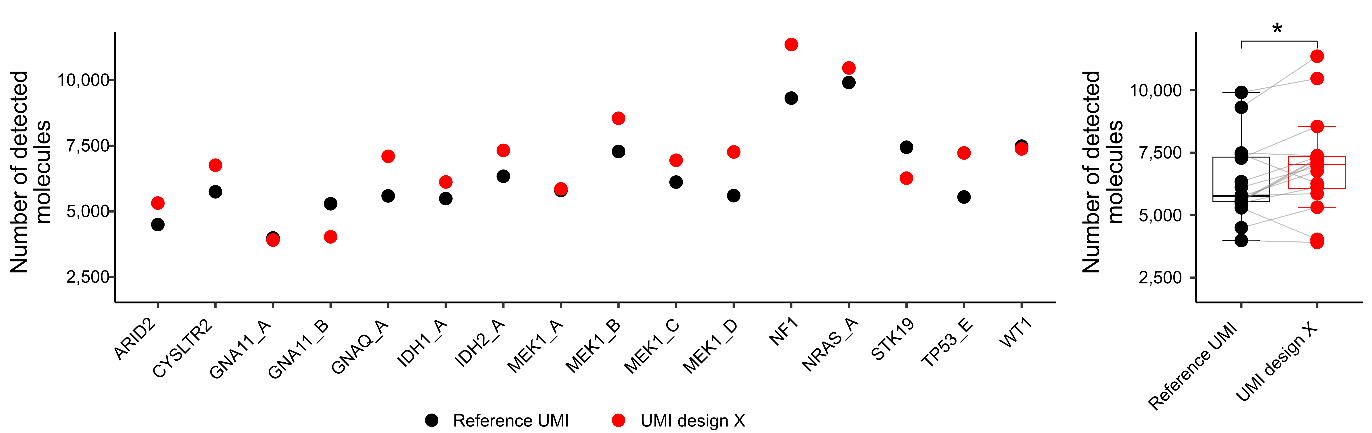


**Fig. S10.** Performance of 16-plex. Number of detected molecules is shown for all individual assays. Mean number is shown for each assay, n = 3. Box plots of all mean values are shown to the right. * p ≤ 0.05, Wilcoxon signed-rank test, n = 16.

**
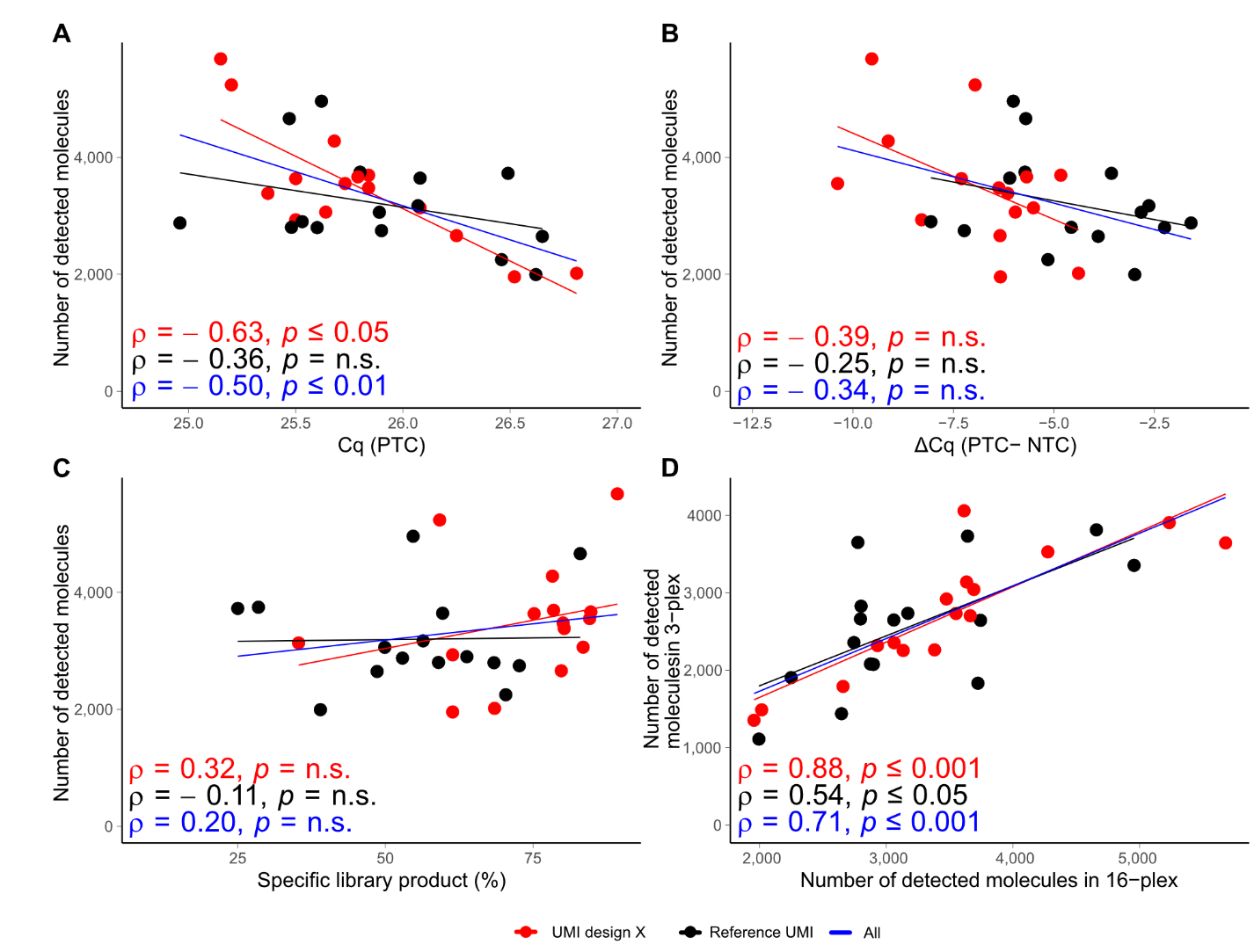
**

**Fig. S11.** Number of detected molecules in a 16-plex in relation to assay performance. **A** The number of detected molecules versus quantitative PCR performance. Number of detected molecules was estimated in the 16-plex data set. Cq is the cycle of quantification value for samples using 20 ng DNA (PTC) determined in the single-plex data set. One cycle corresponds to a two-fold difference in sensitivity to detect target DNA, assuming 100% PCR efficiency and equal fluorescence generation for all assays. **B** The number of detected molecules versus quantitative PCR performance assessed as ΔCq. Number of detected molecules was estimated in the 16-plex data. ΔCq was calculated as the difference in cycle of quantification values between samples using 20 ng DNA (PTC) and no template control (NTC) using the single-plex data set. ΔCq equals one corresponds to a two-fold difference in assay specificity, assuming 100% PCR efficiency. **C** The number of detected molecules versus library purity using parallel capillary electrophoresis. Number of detected molecules was estimated in the 16-plex data, while library purity data was assessed in the single-plex data set. **D** Number of detected molecules of individual assays in tri-plexes versus 16-plex. The Spearman’s correlation coefficients (ρ) are calculated for UMI design X, reference UMI and all together. Each dot corresponds to one individual assay. The linear fits are to guide the eye. NTC, no template control; n.s., not significant.


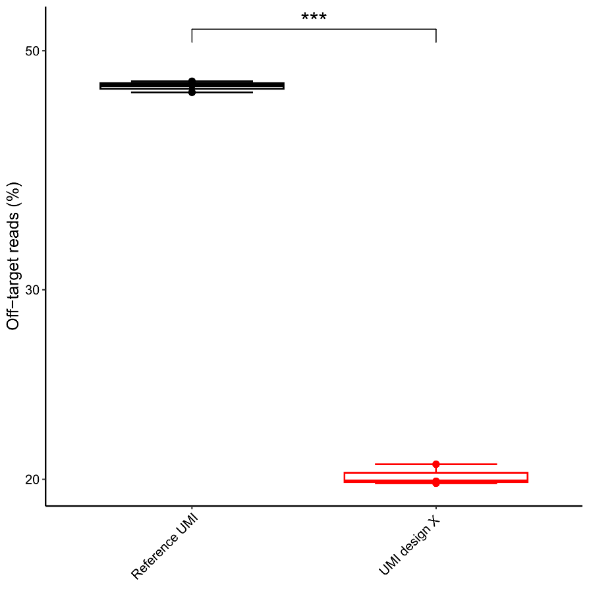


**Fig. S12.** Performance of 16-plex. Mean fraction of off-target reads is shown for the panel, n = 3. *** p ≤ 0.001, unpaired Student’s t-test.

#
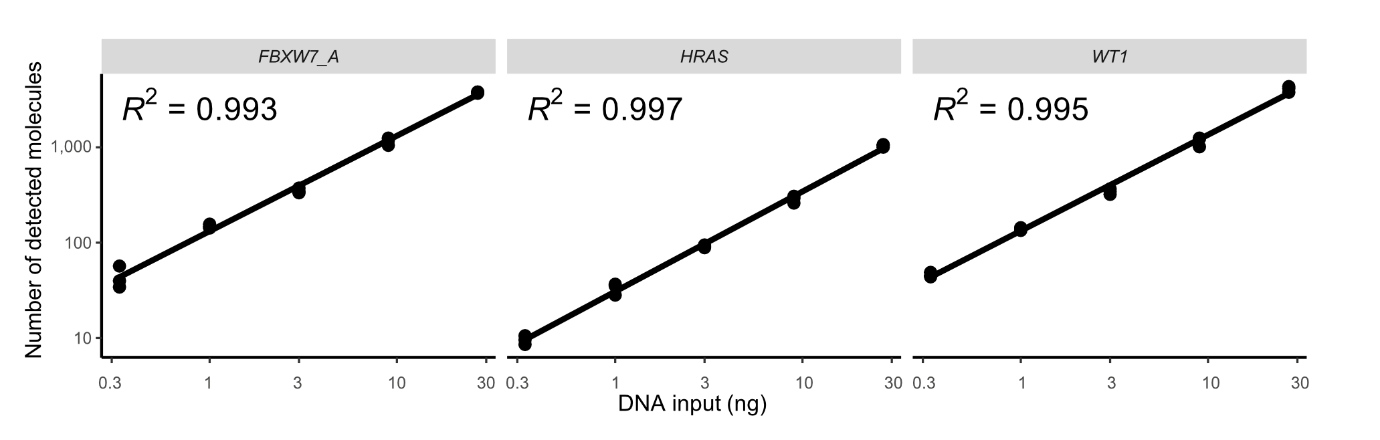


**Fig. S13.** Dynamic range of one tri-plex using UMI design X. Linear regression is shown for each assay, n = 3.


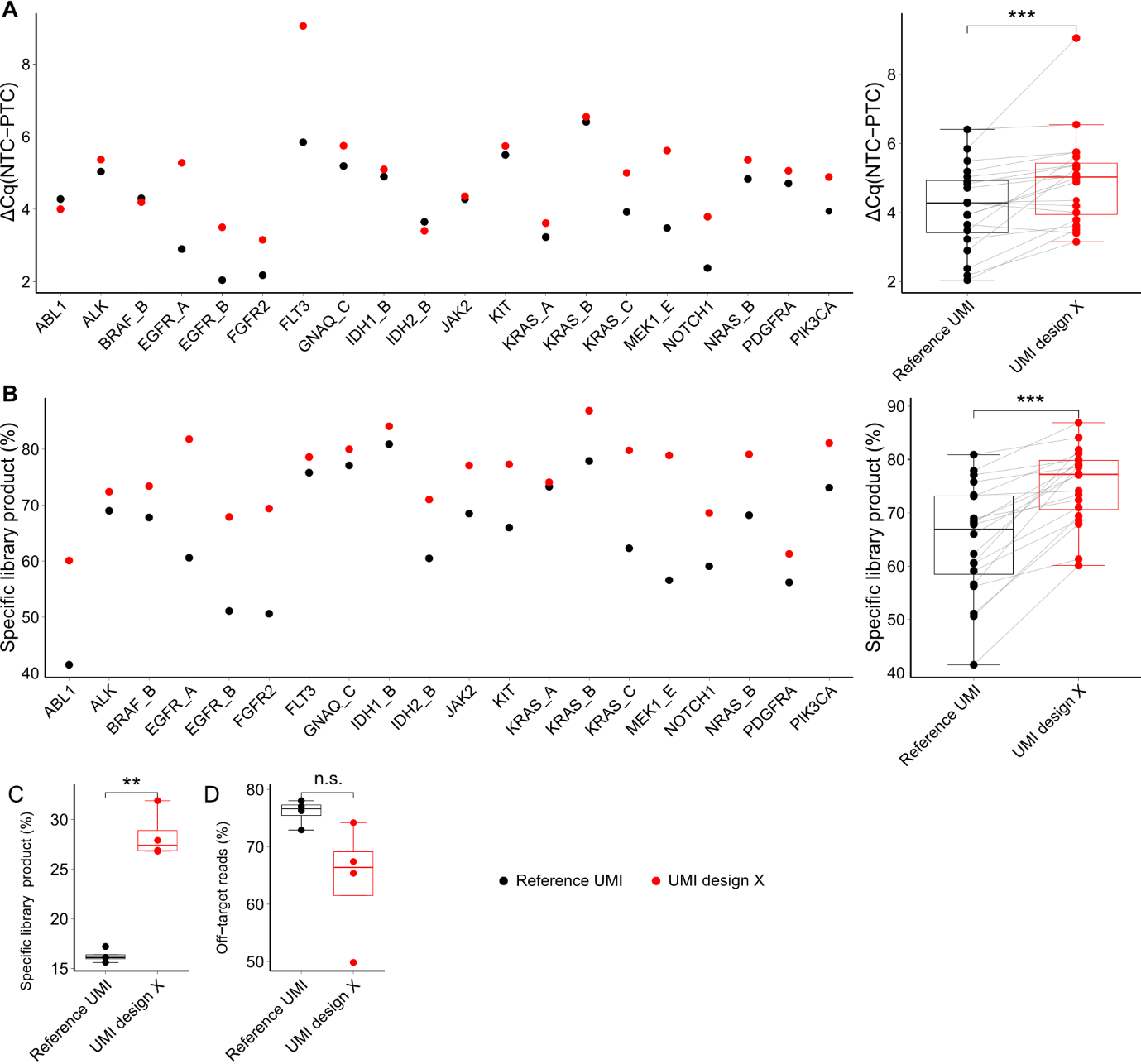
 **Fig. S14.** Hot-spot mutation panel performance. **A** Relative specificity using quantitative PCR. Mean ΔCq was calculated as the difference in cycle of quantification values between samples using 20 ng DNA (PTC) and no template control (NTC). Mean value for each assay is shown n = 3. Box plots of all data are shown to the right. *** p ≤ 0. Wilcoxon signed-rank test, n = 20. **B** Specificity based on correct library product formation using parallel capillary electrophoresis for each assay. The percentage of specific library products relative total DNA amount is shown, n = 1. Box plots of all data are shown to the right. *** p ≤ 0.001, paired Student’s t-test. **C** Specificity based on correct library product formation using parallel capillary electrophoresis for the 20-plex hot-spot mutation panel. The percentage of specific library products relative total DNA amount is shown as box plots, n = 3. *** p ≤ 0.01, unpaired Student’s t-test. **D** Fraction of off-target sequence reads. Box plots are shown, n = 3. n.s., not significant, unpaired Student’s t-test.


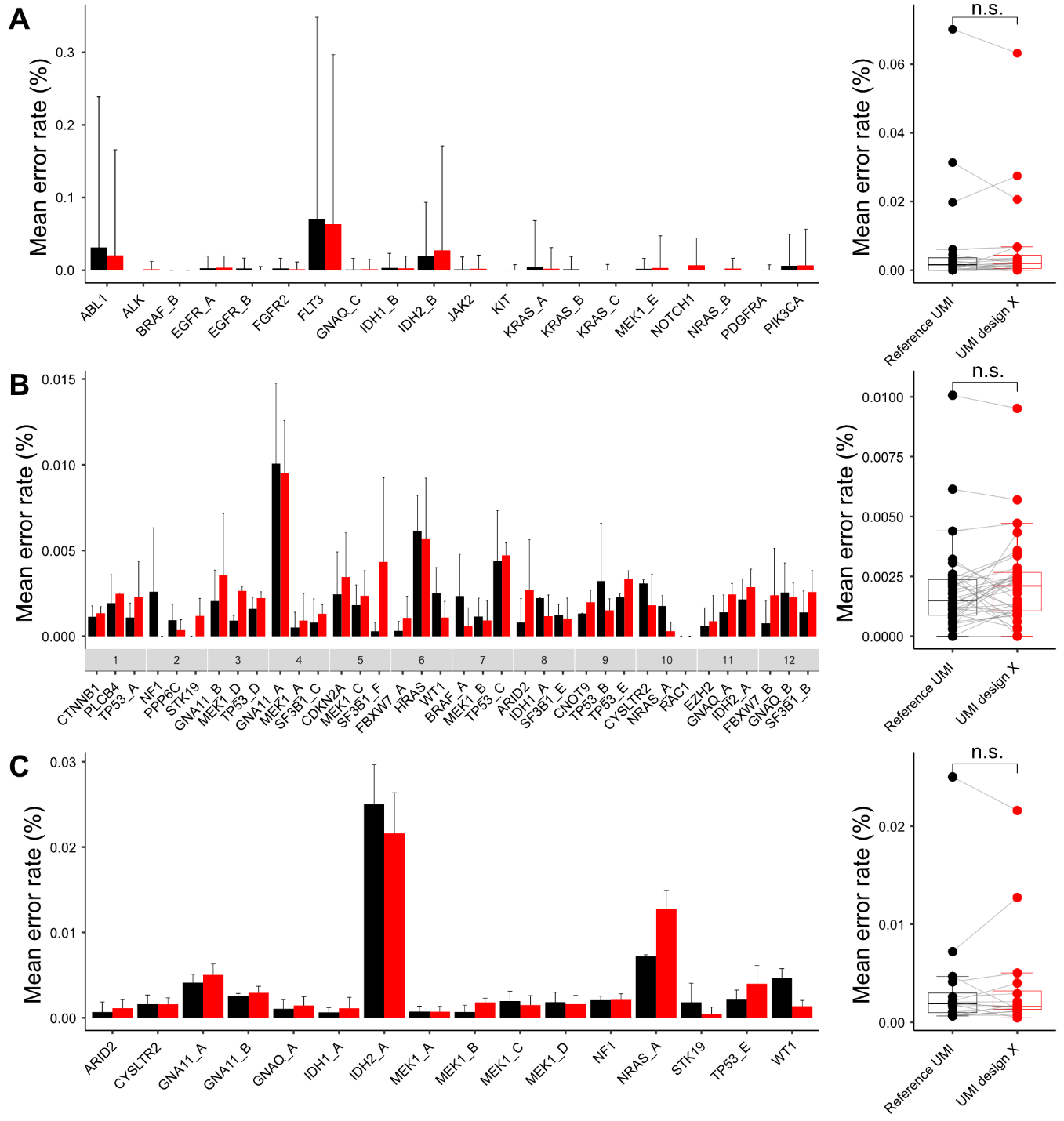


**Fig. S15.** Evaluation of background error rates. **A** Sequencing error rate for hot-spot mutation panel. The error rate was calculated as the total number of non-reference alleles divided by number of molecules at each nucleotide position. Three nucleotide positions corresponding to known single nucleotide variants were omitted for background noise calculations. Box plot of all data is shown to the right. n.s., not significant, Wilcoxon signed-rank test, n = 20. **B** Sequencing error rate for tri-plexes. The mean error rate per amplicon is shown. Three nucleotide positions corresponding to known single nucleotide variants were omitted for background noise calculations. Mean ± S.D. is shown, n = 3. Box plot of all data is shown to the right. n.s., not significant, Wilcoxon signed-rank test, n = 36. **C** Sequencing error rate for 16-plex. Mean ± S.D. is shown, n = 3. Box plot of all data is shown to the right. n.s., not significant, Wilcoxon signed-rank test, n = 16.

**
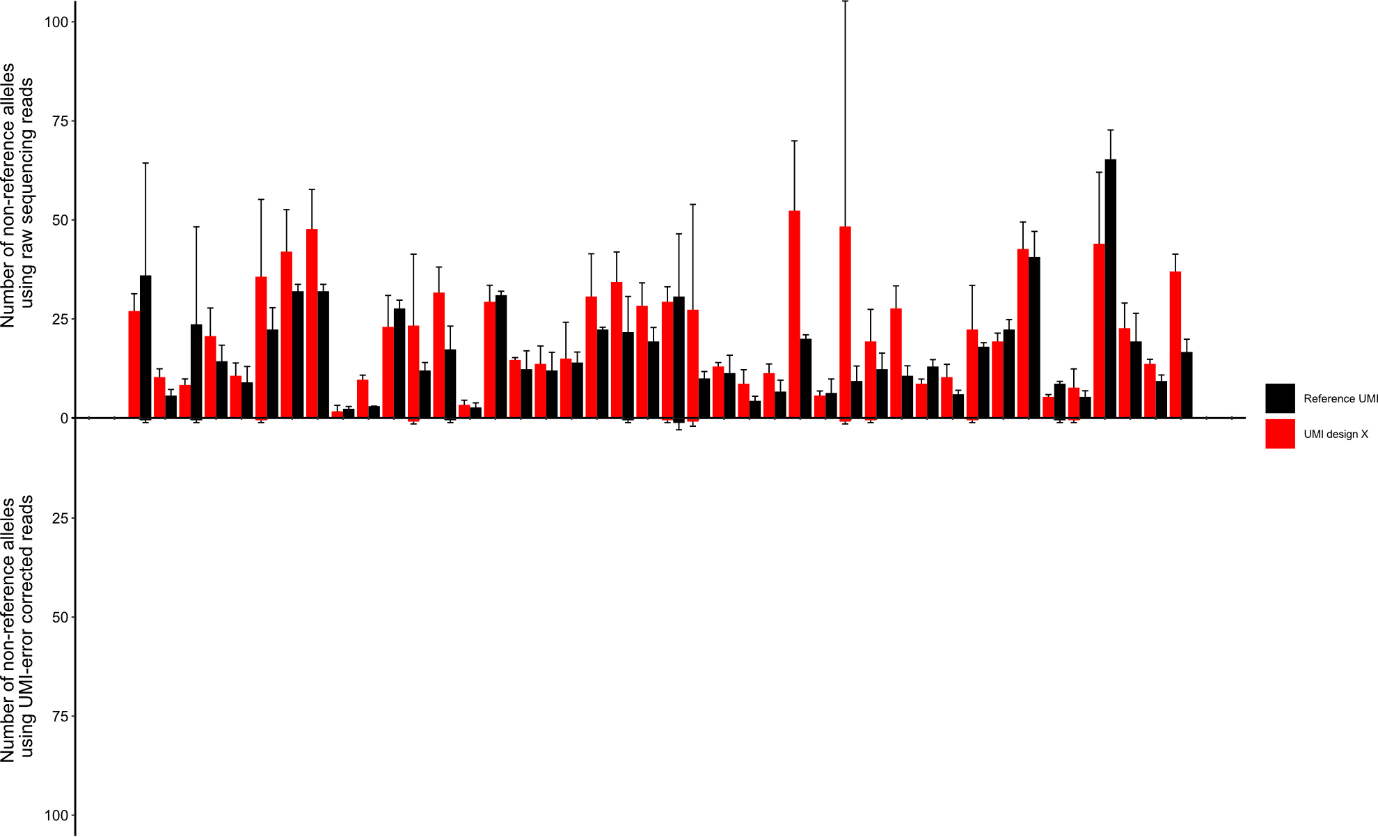
**

**Fig. S16.** Error-correction using UMIs. Example of sequencing data with and without error correction using UMIs for the *CTNNB1* assay. Each bar represents a nucleotide position. Mean ± S.D. is shown, n = 3. Note that the number of non-reference alleles for most nucleotide positions is zero using UMI-error corrected reads.


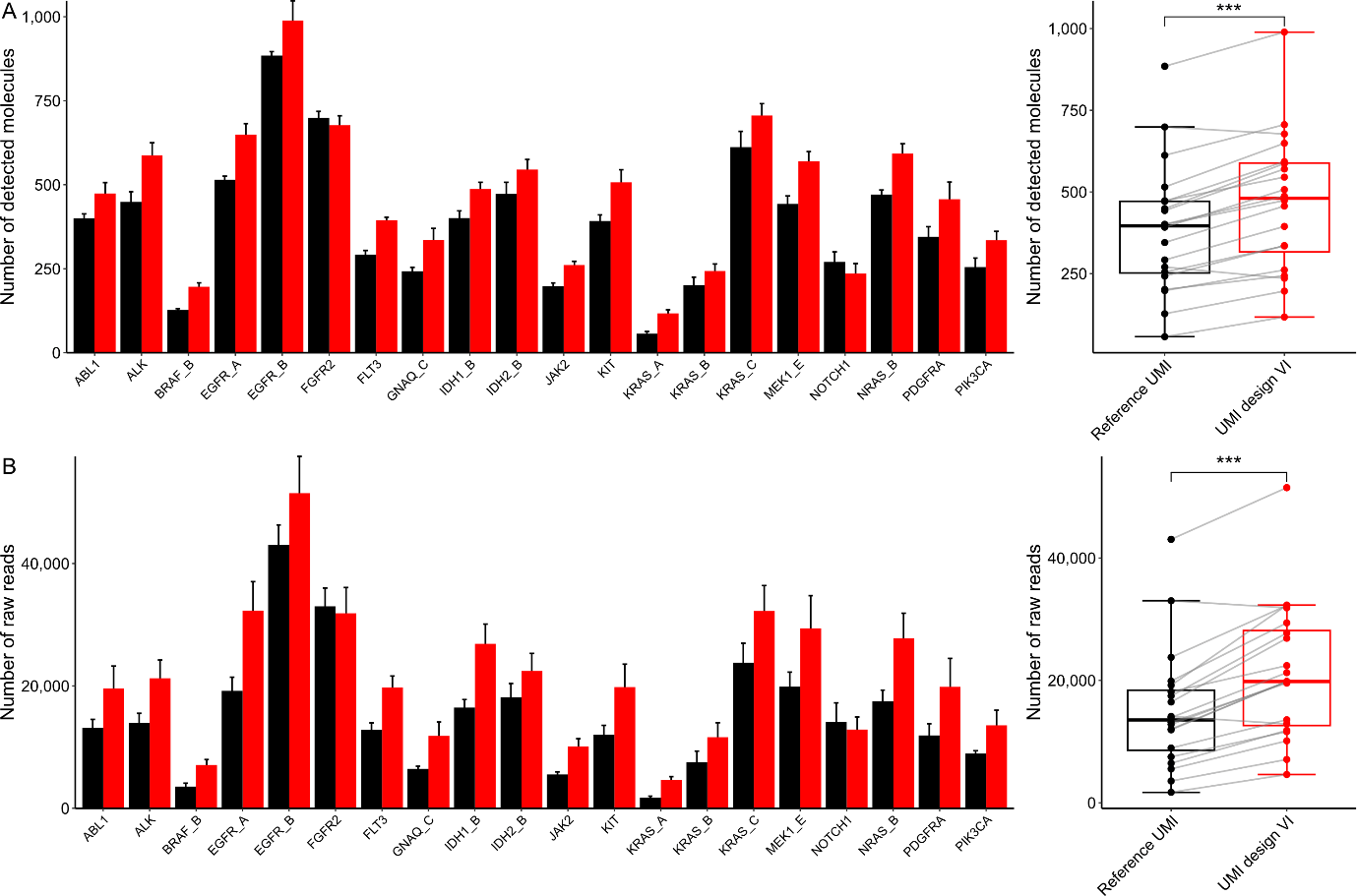
**Fig. S17.** Hot-spot mutation panel performance using sequencing. **A** The number of detected molecules. Mean number for each individual assay is shown, n = 4. Box plots of all mean values are shown to the right. *** p ≤ 0.001, Wilcoxon signed-rank test, n = 20. **B** Number of raw reads. Mean number for each individual assay is shown, n = 4. Box plots of all mean values are shown to the right. *** p ≤ 0.001, Wilcoxon signed-rank test, n = 20.


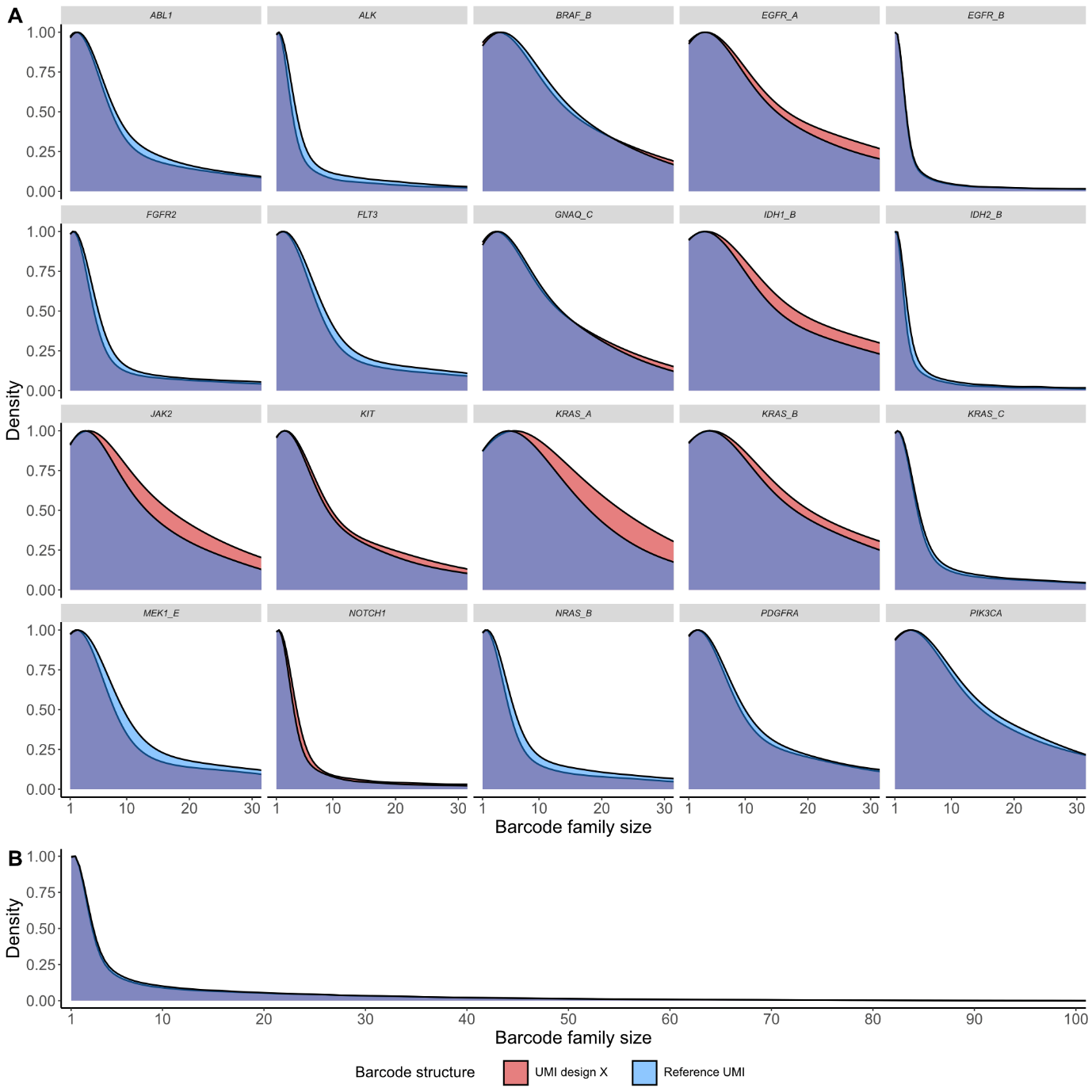


**Fig. S18.** Distribution of UMI-family sizes in hot-spot mutation panel data. **A** Scaled UMI-family sizes per individual assay, n = 4. Purple color indicates overlapping distributions. **B** Scaled UMI-family sizes for the entire hot-spot mutation panel, n = 4.


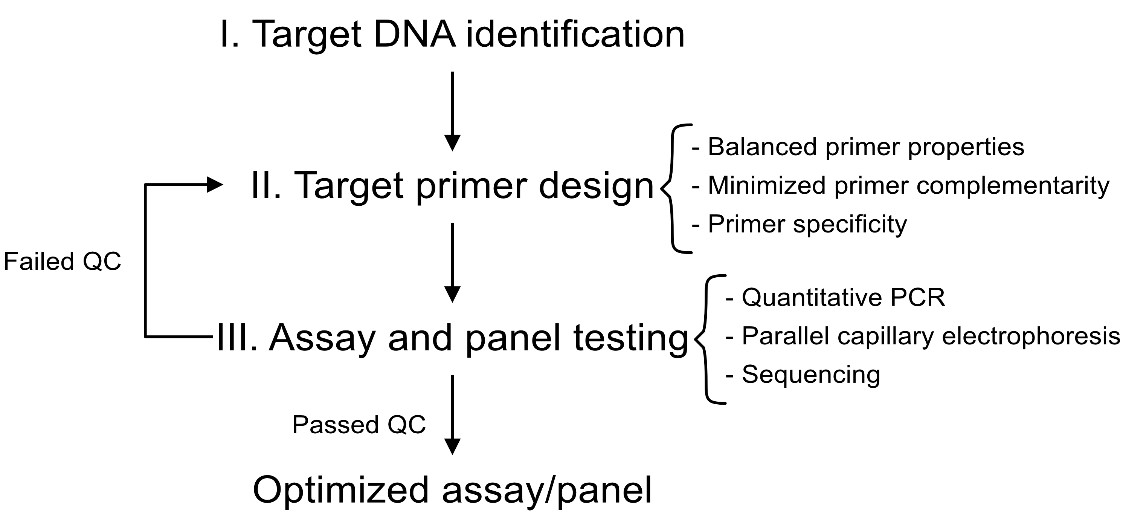
**Fig. S19.** Design and evaluation of SiMSen-Seq assays using structured UMIs.

*Step I. Identify and download target sequences.*

The UCSC Genome Browser (https://genome.ucsc.edu/) can be used. Select ‘Genomes’ in the task bar and choose appropriate genome version. Enter the genomic position of the target sequence, subsequently select ‘View’ in the task bar and choose ‘DNA’. Add 200 base pairs at both the 5′ and 3′ ends, respectively, under ‘Sequence Retrieval Region Options’. Obtain the DNA sequence for the region by pressing ‘get DNA’.

*Step II. Target primer design.*

There are several public and commercial software for primer design, including tools for both single-plex and multiplex designs. Primer-BLAST (https://www.ncbi.nlm.nih.gov/tools/primer-blast/) is an established tool for single-plex design. Here, add the sequence of interest (provided in Step I) into the ‘PCR template’ window. Fill in allowed ‘Range’ so that the target sequence is sandwiched with ≥ 1 base pair gap to the forward and reverse primers, respectively. Use default settings with the following exceptions:

- ‘PCR product size.’ Recommended PCR product size depends on application. For example, cell-free DNA generated from blood plasma is highly fragmented. Hence, the recommended PCR product size is < 80 base pairs.

- ‘Database.’ for human DNA analysis choose ‘Genomes for selected eukaryotic organisms (primary assembly only)’ and Homo sapiens in ‘Organism’.

‘Max Self Complementarity’ and ‘Max Pair Complementarity’ under ‘Advanced parameters.’ To minimize homo- and hetero-dimer formation, set ‘Any’ and ‘3’’, to the values 3 and 2, respectively.

Primer sequences are retrieved by selecting ‘Get Primers’. Observe, often the parameter values need to be relaxed in a stepwise fashion before any primer pair with both high specificity, *i.e.*, no unintended targets of similar size identified by BLAST, and low likelihood of homo- and hetero-dimer formation can be found. For assay multiplexing ‘Primer melting temperatures (T_m_)’ should remain identical for all primer pairs. To complete the primer sequences using UMI design X, add:

5’‑GGACACTCTTTCCCTACACGACGCTCTTCCGATCTNNNNAAANNANNAAANNNNATGGGAAAGAGTGTCC-3’

at the 5’-end of the forward primer and

5’-GTGACTGGAGTTCAGACGTGTGCTCTTCCGATCT-3’

at the 5’-end of the reverse primer. For example, assay *ARID2* (from Additional file 3) with forward target primer:

5’-TGCTCATTTTGAAGTAAATCCAGATTGTTCTGTT-3’

and reverse target primer:

5’-ACTGCAAGTCGAGAGGTATTCAGAATACATT-3’

will become forward barcoding primer:

5’‑GGACACTCTTTCCCTACACGACGCTCTTCCGATCTNNNNAAANNANNAAANNNNATGGGAAAGAGTGTCCTGCTCATTTTGAAGTAAATCCAGATTGTTCTGTT-3’

and reverse barcoding primer:

5’‑GTGACTGGAGTTCAGACGTGTGCTCTTCCGATCTACTGCAAGTCGAGAGGT ATTCAGAATACATT-3’.

Order oligonucleotides and store at -80 ˚C immediately upon arrival. Note that primer quality may vary substantially between manufacturers and batches. Here, we used Ultramers from Integrated DNA Technologies.

*Step III. Assay and panel evaluation.*

Assays and panels should be experimentally evaluated. Individual assays and small multiplexes (≤ 3 assays) benefit from evaluation with quantitative PCR after the initial barcoding PCR step (Fig. 2A). The cycle of quantification (Cq) value of DNA positive samples is a measure of PCR efficiency. If the Cq value is lower than that of a well-performing reference assay it implies poor specificity, while a high Cq value suggests low PCR efficiency. ΔCq is calculated as the difference between samples with and without DNA, where a ΔCq value > 2 using 20 ng DNA as starting material indicates a well-performing assay.

Both individual assays and multiplexed panels may be evaluated using parallel capillary electrophoresis to assess library yield and specificity, following the barcoding and adapter PCR steps and prior to library purification (Fig. 2B). Library purity is calculated as the fraction of specific library product compared with total DNA amount. For amplicon sizes ranging 60-100 base pairs, we recommend that yield and purity are based on the 210 - 300 base pairs interval. For example, the *ARID2* assay has a 76 base pairs amplicon, consequently the SiMSen-Seq library will be 233 base pairs using UMI design X. A well-performing single-plex assay generally yields > 25% specific library product. For larger multiplex panels, the specific yield is expected to decrease but still perform adequately in sequencing.

Sequencing will provide the most detailed information about assay and panel performance. Metrics include sensitivity, specificity, error rate and panel uniformity. The sensitivity can be assessed by comparing the amount of detected molecules in relation to input DNA. Specificity can be determined by the degree of off-target reads as the sum of all reads not correctly aligned. The error rates are generally minimal, where error rates << 0.1% are expected at essentially all nucleotide positions. The expected degree of uniformity between assays in a panel is highly target primer and amplicon dependent. The assay uniformity shown for the hot-spot mutation panel in Additional file: Fig. S17A serves a relevant example of a typical panel.

From a practical point of view, quantitative PCR is fast and easy to perform followed by parallel capillary electrophoresis. Sequencing provides the most informative evaluation but requires more time and resources to perform. Hence, the degree and type of assay and/or panel validation depends on application and analytical requirements. If assays and/or panels pass the outlined quality controls (QC), they are suitable for digital sequencing using the SiMSen-Seq approach. If assays fail the quality controls, target primers need to be redesigned and retested.


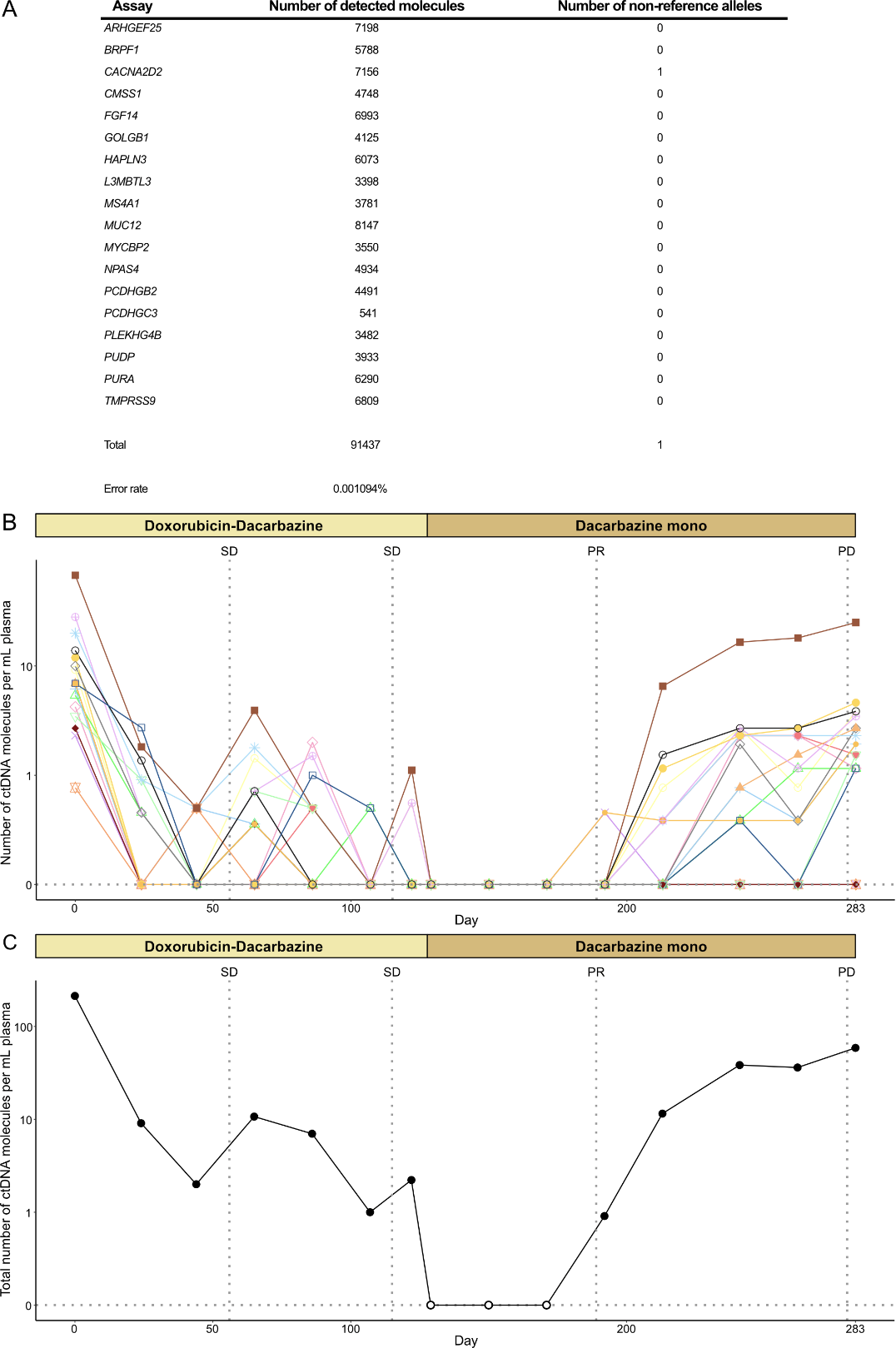


**Fig. S20.** Circulating tumor-DNA analysis in leiomyosarcoma. **A** Error-rate for the personalized ctDNA panel. **B** Number of ctDNA molecules per mL plasma for 18 patient-specific mutations during palliative chemotherapy in a patient diagnosed with leiomyosarcoma. Plasma samples were analyzed with a personalized ctDNA panel using SiMSen-Seq with UMI design X. Treatments and results from radiological evaluations are shown at the top of the diagram. The number of ctDNA molecules is shown in log_10_-scale. PR, partial response; SD, stable disease; PD, progressive disease. **C** Total number of ctDNA molecules per mL plasma. Treatments and results from radiological evaluations are shown at the top of the diagram. Open circles represent no detectable ctDNA. The total number of ctDNA molecules is shown in log_10_-scale.


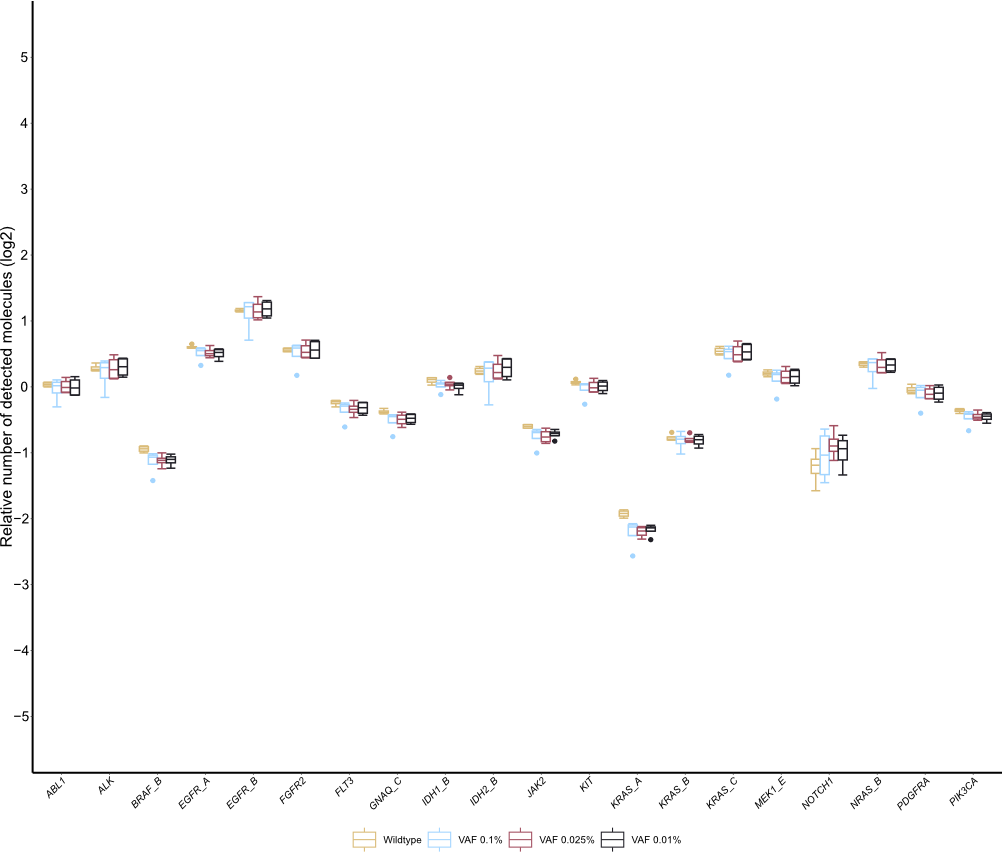


**Fig. S21.** Assay performance in hot-spot panel. The relative number of detected molecules per assay and sample group is shown. Data are mean-centered around the overall mean of 5030 molecules. Box plots of all mean values are shown, n = 4.
