# Supplementary material for: Digital sequencing is improved by using structured unique molecular identifiers
Source: Genome Biol. 2025 Feb 25;26:37. doi: 10.1186/s13059-025-03504-x (PMC11853513; doi:10.1186/s13059-025-03504-x)
Supplement: Supplementary file 6 — Additional file 6. Peer review history. [file 13059_2025_3504_MOESM6_ESM.docx]

**Review history**

**First round of review**

**Reviewer 1**

This paper is a clear and well-constructued report, demonstrating convincingly that DNA molecular barcoding, an essential step in many applications of DNA seqeuncing, can be substantially improved by the use of structured bardcodes, with some concrete examples of such barcodes, and the improvements they yield. A limitation of the study is that it only considers the influence of the structured barcodes in the context of one specific method, SiMSen-seq. I think this should be acknowledged up-front, as no evidence is presented that the strctured barcodes would add value in any other context.
The only other comment is that, at least in my version, the figures were of low resolution; specifically I was unable to read the seqeunces of the different barcodes in Fig1C/D. This information is core to the paper, so needs to be very clear and legible.

**Reviewer 2**

The authors argue that one issue with PCR-based library construction is the generation of non-specific PCR products due to random UMIs, which adversely affect the performance of these methodologies. To combat this, the authors have integrated a novel unstructured UMI design into the established SiMsen-Seq technology. This innovation entails the incorporation of predefined nucleotides at specific positions within the UMI, leading to a marked increase in specificity and sensitivity in the experimental setups used, including separate assays and multiplexed panels. While the advance is beneficial for the field, the paper could be significantly enhanced by elucidating the rationale behind the UMI design principles and contextualizing the solution within a biological question. Additionally, a more comprehensive benchmarking of the structured UMI designs, along with a computational tool—or at least detailed guidelines—for designing UMIs for specific targets would significantly augment the study's value.

Comments

Major

1. The manuscript is generally well-written and logical, but at times, it reads like a methods section. The initial experimental setup requires a more detailed description and justification. Specifically, what is the foundational rationale for the proposed structured UMI designs (see Figure 1C,D)? Were these designs informed by systematic computational modeling? This section would benefit from more quantitative comparisons, such as contrasting the proposed sequence characteristics with reference sequences.

2. From the analyses conducted, the authors favored UMI design VI for further analysis due to its higher complexity, despite UMI III demonstrating superior specificity. A concern arises from the choice of UMI VI, as the variability in measured data is not addressed. Notably, UMI VI exhibits a bimodal distribution in relative specificity scores, indicating that it did not offer a consistent advantage in at least two out of six assays (see Figure 2C). It would have been advantageous from an assay development perspective to further refine UMI III to enhance complexity and optimize detection efficiency. The omission of such optimization reduces the conceptual impact of the paper.

3. The latter sections of the manuscript (Figures 3-5) delve deeply into the technical aspects and lose sight of the biological question at the heart of this research. The primary goal of UMI-incorporated PCR-based library construction methods, including SiMsen-Seq, is to enhance the detection of rare targets with variant allele frequencies below 0.1%, a current challenge in the field. The authors would add significant value to the study by broadening the gene/sample spectrum to assess the efficiency of their UMI design, particularly at these lower allele frequencies. Additionally, the performance of UMI design VI appears inconsistent across the assays. What are the reasons for this variability? Are some gene subsets more amenable to this UMI design than others, and could alternative designs be more effective for different gene subsets (see also comment above)? A deeper analysis and discussion regarding the performance variations with the chosen UMI design would be informative.

4. The rationale for designing structured UMIs is to diminish the production of non-specific PCR products, thereby improving the performance of methods like SiMsen-Seq. Yet, this assertion remains ambiguous as it does not concretely show the impact of these non-specific products on performance. It would be instructive to clarify the extent of this interference, for instance, whether it affects the variant allele frequency (VAF) detection limit of SiMsen-Seq or other aspects. Accordingly, the benchmarking criteria should hone in on enhancing the detection threshold of SiMsen-Seq.

5. To maximize the impact of their work and provide a significant service to the research community, the authors should offer a computational tool or at least a detailed set of guidelines to aid in the creation of custom UMI designs.

Minor

1. The term "digital sequencing" used in the manuscript is somewhat confusing; it is not commonly used in this context. The term 'digital' typically applies to droplet digital PCR (ddPCR), which involves the individual measurement of DNA molecules in discrete droplets. SiMsen-Seq, although sensitive in quantifying mutations, does not operate on the same principle as ddPCR, making the term 'digital sequencing' potentially misleading.

2. The introduction would benefit from a more comprehensive literature review that places this study in the context of existing methodologies, such as the one detailed by Peng et al., Scientific reports 2019 (https://www.nature.com/articles/s41598-019-41215-z). It should also provide a more thorough explanation of the SiMSen-Seq pipeline and its modifications that form the basis for this paper's improvements.

3. A consolidated table summarizing the metrics tested would offer readers a clearer synthesis of the results. Additionally, expanding the Supplementary material to include raw data and a more detailed data description would greatly aid the paper's transparency and utility.

4. In Figures 3-5, would it be possible to apply a non-parametric rank test instead of the Student's t-test to accommodate data normality concerns?

5. There appears to be a display issue with Supplementary Figure 7 that needs correction.

**Authors’ response to reviewers**

**Reviewer #1:** This paper is a clear and well-constructed report, demonstrating convincingly that DNA molecular barcoding, an essential step in many applications of DNA sequencing, can be substantially improved by the use of structured barcodes, with some concrete examples of such barcodes, and the improvements they yield. A limitation of the study is that it only considers the influence of the structured barcodes in the context of one specific method, SiMSen-seq. I think this should be acknowledged up-front, as no evidence is presented that the structured barcodes would add value in any other context.

The only other comment is that, at least in my version, the figures were of low resolution; specifically I was unable to read the sequences of the different barcodes in Fig1C/D. This information is core to the paper, so needs to be very clear and legible.

*- We thank the reviewer for the positive feedback and useful comments. We have updated the manuscript and pointed out that we used the SiMSen-Seq approach in the Abstract and elsewhere. We have also increased the font size in Figures 1C-D. The original figure was submitted in high resolution and we believe the low resolution is a result of the file-format that is provided to the reviewer. We also provide all sequences in Additional file 3, highlighted in the legend for Figure 1.*

**Reviewer #2:** The authors argue that one issue with PCR-based library construction is the generation of non-specific PCR products due to random UMIs, which adversely affect the performance of these methodologies. To combat this, the authors have integrated a novel unstructured UMI design into the established SiMsen-Seq technology. This innovation entails the incorporation of predefined nucleotides at specific positions within the UMI, leading to a marked increase in specificity and sensitivity in the experimental setups used, including separate assays and multiplexed panels. While the advance is beneficial for the field, the paper could be significantly enhanced by elucidating the rationale behind the UMI design principles and contextualizing the solution within a biological question. Additionally, a more comprehensive benchmarking of the structured UMI designs, along with a computational tool—or at least detailed guidelines—for designing UMIs for specific targets would significantly augment the study's value.

*- We thank the reviewer for the positive and constructive feedback, contributing to an improved manuscript. We have included several figures with additional in-depth information and new analyses, shown in Additional file 1: Supplementary Figure 1-4, 7 and 16 (we are open to include them in the main manuscript if recommended). We have also made a publicly available computational tool to simulate the probability of unintended UMI (*[*https://github.com/stahlberggroup/structuredUMI*](https://github.com/stahlberggroup/structuredUMI)*).*

Comments

Major

1. The manuscript is generally well-written and logical, but at times, it reads like a methods section. The initial experimental setup requires a more detailed description and justification. Specifically, what is the foundational rationale for the proposed structured UMI designs (see Figure 1C,D)? Were these designs informed by systematic computational modeling? This section would benefit from more quantitative comparisons, such as contrasting the proposed sequence characteristics with reference sequences.

*- This is an insightful comment. There are several aspects of the structured UMI designs. Several are from accumulated experience working with randomized sequences and degenerative oligonucleotides in applications related to PCR, qPCR, digital PCR, sequencing and digital sequencing. We performed some simulations related to UMI-UMI interactions. Here UMI design V – VIII and XIII performed best (new Supplementary Figure 4A). The rationale behind the UMI designs is now explained in more detail (Lines 101-137). We have also uploaded the simulation tool to study UMI-UMI interactions at Github (*[*https://github.com/stahlberggroup/structuredUMI*](https://github.com/stahlberggroup/structuredUMI)*) since it may be an informative tool for different UMI designs and methods. The information about the simulation of UMI-UMI interaction is descried in Methods (Lines 449-455). However, all experimental parameters during library construction that affects interactions and performance are not possible to model, i.e., advanced primer modeling tools are only able to handle target primers. This is also confirmed by our simulations where some UMI designs (like UMI design III) should potentially perform worse than the reference UMI. However, this was not experimentally observed, indicating that UMI-UMI interactions are not the major source of non-specific PCR products (Lines 163-166). In conclusion, our data clearly indicate that it is the use of structured UMIs in combination with the experimental protocol that together result in superior performance. We also extended our Discussion about the UMI designs in relation to our experimental data (Lines 321-331).*

*Another aspect of the structured UMI design is to either minimize or increase the internal interactions between the UMI and other parts of the oligo. The ability to form an additional hairpin and/or stem structure within the loop structure is addressed by the UMI designs to different degrees. Here, structured UMI designs I-XIII should all reduce the interactions with the adapter and stem sequences, while design XIV instead intentionally may form an additional stem (see new Supplementary Figure 2B). The idea of the last design was to observe if a more closed loop structure would outcompete other UMI interactions. For example, to minimize the risk that the target primer sequence of either primer bind to the UMI as a hairpin (see new Supplementary Figure 2E). The new Supplementary Figure 2A-G schematically illustrates the potential issues with the UMI in relation to intended and unintended interactions. We have now also updated the manuscript with a step-by-step guideline how to design SiMSen-Seq assays (new Supplementary Figure 16, see response to comment 5 for more details).*

*After the initial UMI design screen (experimental data in Figure 2), it become clear that all structured UMIs were potentially useful in comparison to the unstructured reference UMI. The two UMI designs that showed smallest improvements in performance were UMI design XIV, with the additional internal stem, and UMI design I, the design that was most similar to reference UMI. The improvements in performance of other UMI designs were larger compared to the reference UMI. However, the differences between the top UMI designs were small. We preferred UMI design VI over UMI design III, since it has eight times higher diversity, which in principle enables this design to be used with more input DNA (see Supplementary Figure 3 and response to comment 2 for more information about the importance of UMI diversity).*

2. From the analyses conducted, the authors favored UMI design VI for further analysis due to its higher complexity, despite UMI III demonstrating superior specificity. A concern arises from the choice of UMI VI, as the variability in measured data is not addressed. Notably, UMI VI exhibits a bimodal distribution in relative specificity scores, indicating that it did not offer a consistent advantage in at least two out of six assays (see Figure 2C). It would have been advantageous from an assay development perspective to further refine UMI III to enhance complexity and optimize detection efficiency. The omission of such optimization reduces the conceptual impact of the paper.

*- Indeed, in Figure 2C, Design VI has a biomodal expression pattern. However, all six assays improved using UMI Design VI compared with reference UMI (see Additional file 4 for individual values). The bimodality was not observed for the parallel capillary electrophoresis data in figure 2D using the same assays. Furthermore, in the follow-up data in Figure 3 with addition of 36 assays, we do not observe any bimodality for UMI Design VI. The rationale of using UMI design VI is based on maintained UMI diversity, to keep the same diversity we would need to extend UMI Design III with four extra nucleotides. Note that UMI Design III is already two nucleotides longer than UMI Design VI. Our new simulations show that the UMI-UMI interactions decrease with UMI design VI, while they increase with UMI design III. To illustrate the importance of UMI diversity in digital sequencing we have generated and included probability data about UMI collisions (new Supplementary Figure 3). We have updated the manuscript with additionally motivation for choosing UMI Design VI over UMI III in relation to UMI diversity (Lines 166-168). We have also included additional information about the importance retaining high UMI diversity in the UMI design (Lines 130-132). If the UMI diversity decreases the chance that a single mutant molecule will be labeled with the same UMI as another DNA molecule increases. Similarly, the chance that two or more DNA molecule will be labeled with the same UMI increases. Supplementary Figure 3 clearly demonstrating the difference between the UMI designs with highest and lowest diversity. Furthermore, our new data based on simulations also add additional information regarding the optimal design (Supplementary Figure 4).*

3. The latter sections of the manuscript (Figures 3-5) delve deeply into the technical aspects and lose sight of the biological question at the heart of this research. The primary goal of UMI-incorporated PCR-based library construction methods, including SiMsen-Seq, is to enhance the detection of rare targets with variant allele frequencies below 0.1%, a current challenge in the field. The authors would add significant value to the study by broadening the gene/sample spectrum to assess the efficiency of their UMI design, particularly at these lower allele frequencies. Additionally, the performance of UMI design VI appears inconsistent across the assays. What are the reasons for this variability? Are some gene subsets more amenable to this UMI design than others, and could alternative designs be more effective for different gene subsets (see also comment above)? A deeper analysis and discussion regarding the performance variations with the chosen UMI design would be informative.

*- We apologize for this confusion, it is our mistake not clearly indicating that this manuscript should primarily be considered as a Method paper (also pointed out by the Editor). We have updated the manuscript according to the Method layout for the journal (for example the Abstract should be < 100 words and manuscript should include a Conclusion section.*

*All digital sequencing based on UMIs have similar theoretical ability to detect low variant allele frequencies, since the polymerase-induced errors are bioinformatically, not experimentally, corrected by consensus read formation (for more details, please see our recent review about digital sequencing methods using UMIs, see PMID:* 38367531*). The main differences between these methods are how they add the UMI to the target sequence and their ability of multiplexing. We have extended the Background with more information, about different methods (Lines 61-67). As expected, we do not observe any effects on error-correction using structured UMIs compared with reference UMI (Supplementary Figures 12 and 13).*

*We believe that the more informative parameter to assess different digital sequencing approaches with each other is the sensitivity to detect target DNA molecules. The major merits of structured UMIs are their improved experimental properties, resulting in increased sensitivity to detect molecules. This is especially important in all applications with limited amount of DNA, such as liquid biopsies and analysis of cell-free DNA. Here, many scientific studies report variant allele frequency for UMI-based methods. However, more and more studies start to report number of mutant molecules per mL plasma instead. We acknowledge that some digital sequencing approaches cannot generate absolute counts. Here, other indirect measures are needed.*

*The improved assay performance of structured UMIs also results in less design and optimization work when designing assays and panels, something that is important in personalized mutation panels that are commonly used for mutation detection using liquid biopsies collected from cancer patients as well as allow optimal use of sequencing capacity. All in all, structured UMIs help reducing costs.*

*UMI Design VI was superior to reference UMI for most assays. All assays are expected to perform differently due to the target primer sequences and the sequence context of entire target amplicon. SiMSen-Seq assays will never perform better than primers containing only target sequence. Hence, similar variability is observed for any well-performing PCR-based digital sequencing method. To study the effects of target primer sequences in more detail, we compared our assay performance data with the ability of primers to form homo- and hetero-dimers as well as to their GC contents (new Supplementary Figure 7 and Lines 187-190). However, we observed no trends, not for UMI design VI nor for reference UMI in relation to these parameters. Another factor that complicates the development of assays is errors in chemical oligonucleotide synthesis. These errors are sequence context dependent, batch-dependent as well as related to chemistry and purification strategy. The consequence is that a significant number of primers have errors (both deletions and substitutions) that may affect assay performance dramatically. For more information about chemical oligonucleotide synthesis errors, please see PMID:*34459892*. This problem is also discussed in the manuscript (Lines 356-358).*

*To demonstrate the use of structured UMIs in a clinically relevant panel we designed a generic hot-spot mutation panel. We used standardized reference material (from Horizon) that are commonly used to benchmark different methods. Our panel is designed with short amplicons to make it also suitable for cell-free DNA analysis. Hence, our current hot-spot mutation panel can be of used by others in various applications. We have now clarified that this panel target clinically relevant mutations and can be used for cell-free DNA analysis (Lines 251-254). It is beyond the scope of this Method article to apply the panel to sample cohorts, demonstrating clinical relevance.*

4. The rationale for designing structured UMIs is to diminish the production of non-specific PCR products, thereby improving the performance of methods like SiMsen-Seq. Yet, this assertion remains ambiguous as it does not concretely show the impact of these non-specific products on performance. It would be instructive to clarify the extent of this interference, for instance, whether it affects the variant allele frequency (VAF) detection limit of SiMsen-Seq or other aspects. Accordingly, the benchmarking criteria should hone in on enhancing the detection threshold of SiMsen-Seq.

*- We show that structured UMIs significantly improve the sensitivity to detect molecules, especially rare mutant molecules compared with reference UMIs, i.e., limit of detection. Here, we demonstrate superior sensitivity to detect molecules, including rare, mutated molecules when benchmarking structured UMI design VI to reference UMI using the SiMSen-Seq approach. As outlined in response to comment 3 above, we argue that sensitivity – in the sense of detecting absolute number of molecules – is the most relevant parameter to assess when identifying rare mutated DNA molecules. The use of UMIs, regardless of method, will bioinformatically correct polymerase-induced errors that affect the ability to detect low variant allele frequency. Next, we demonstrate reduced off-targets reads, which significantly reduce the need of sequencing capacity and costs. Finally, we argue that improved assay performance will reduce the design and optimization time for new panels, since more assays will pass user-defined validation criteria (see Supplementary Figure 16 for our recommendations).*

*We demonstrate improved assay performance using several different metrics, including quantitative PCR, parallel capillary electrophoresis and sequencing (see new Table 1 for overview). All these methods provide several output parameters that demonstrate different assay properties. Importantly, all metrics demonstrate significant and systematic improvements when comparing structured UMI with unstructured reference UMI. Here, we focus on UMI design VI, but our screen indicates that essential all structures UMIs are potentially beneficial, indicating that structured UMIs should be considered in other UMI-based approaches as well. Please, see our response to Reviewer comment 3 for additional information.*

5. To maximize the impact of their work and provide a significant service to the research community, the authors should offer a computational tool or at least a detailed set of guidelines to aid in the creation of custom UMI designs.

*- We thank the reviewer for this comment. We have now updated the manuscript with a detailed guideline how to design assays and panels as well as how to evaluate them, which will help end-users apply SiMSen-Seq with structured UMIs (new Supplementary Figure 16 with extended legend, Lines 285-287). We are now also providing a simulation tool to evaluate UMI-UMI interactions (see also response to Reviewer comment 1). We have also extended the rationale for our designs and the importance of maintaining high diversity (Lines 101-137 and new Supplementary Figure 3).*

Minor

1. The term "digital sequencing" used in the manuscript is somewhat confusing; it is not commonly used in this context. The term 'digital' typically applies to droplet digital PCR (ddPCR), which involves the individual measurement of DNA molecules in discrete droplets. SiMsen-Seq, although sensitive in quantifying mutations, does not operate on the same principle as ddPCR, making the term 'digital sequencing' potentially misleading.

*- We agree to some degree, the word digital refer to the ability to analyze molecules one by one. For PCR-based methods, we use digital PCR to count individual molecules. In some UMI- and sequencing-based approaches, such as the one used here, we also look at one molecule at the time where we collapse the raw sequencing reads to consensus reads. Only if a molecule is observed three or more times (note: a threshold that can be increased or decreased) we use it for downstream analysis, others are discarded. Correspondingly, even if we see the same molecule 100 times, we only count it once. Hence, we believe that referring to our approach as ‘digital sequencing’ still is relevant since it is conceptionally very similar to digital PCR. However, we are open to change the wording if requested.*

2. The introduction would benefit from a more comprehensive literature review that places this study in the context of existing methodologies, such as the one detailed by Peng et al., Scientific reports 2019 (<https://www.nature.com/articles/s41598-019-41215-z>). It should also provide a more thorough explanation of the SiMSen-Seq pipeline and its modifications that form the basis for this paper's improvements.

*- There are numerous methods based on standard UMIs, in addition to SiMSen-Seq. For an overview of UMI-based methods, please see the recent review PMID:* 38367531 (now included in Background)*. However, none of these approaches are based on structured UMIs, so the experimental improvements using structured UMIs is not previously described to our knowledge. We have updated and extended the Background information about digital sequencing approaches, highlighting the differences and examples of methods (including the useful references suggested here) in the two major groups: PCR-based methods and hybridization capture-based approaches (Lines 61-67).*

*We have also outlined the concept of UMIs and the bioinformatics in SiMSen-Seq (new Supplementary Figure 1, Lines 70-71). In addition, we have provided a detailed step-by-step guideline how to design and evaluate SiMSen-Seq assays and panels (new Supplementary Figure 16, Lines 285-287). This new information together with the library construction details outlined in Figures 1A-B and Lines 91-100 provide a comprehensive overview of all aspects related to SiMSen-Seq, including the use of structured UMIs.*

3. A consolidated table summarizing the metrics tested would offer readers a clearer synthesis of the results. Additionally, expanding the Supplementary material to include raw data and a more detailed data description would greatly aid the paper's transparency and utility.

*- Good suggestion. We have included a summarizing Table 1 (Line 86). We have also included all source data for all figures as Additional files 4 and 5 to increase the transparency of all data and plots.*

4. In Figures 3-5, would it be possible to apply a non-parametric rank test instead of the Student's t-test to accommodate data normality concerns?

*- Agreed, we have updated the statistics in Figures 3-5 with non-parametric rank tests.*

5. There appears to be a display issue with Supplementary Figure 7 that needs correction.

*- Supplementary Figure 7 (now supplementary Figure 12) is actual correct. The number of non-reference alleles after UMI-error correction are few, for most nucleotide position we cannot detect any background noise. We have pointed this out this in the legend since it was confusing.*

**Second round of review**

**Reviewer 1**

No further comments

**Reviewer 2**

We are pleased to see the improvements in this manuscript, particularly the addition of Supplementary Figures 1 and 2, which clarify the rationale behind the UMI design. The inclusion of a step-by-step guideline (Supplementary Figure 16) and computational tools to simulate the probability of unintended UMIs are also valuable additions. Despite these efforts, the revised manuscript provides seemingly limited additional value compared to the original version though. Beyond the benchmarking experiment, the authors should in our opinion demonstrate that the new UMI design can address challenges that cannot be met by current strategies. Without such experimental proof, even if only a proof-of-concept, it remains difficult to assess the significance of this paper and its impact on the field.

Major comments:
1. The structured UMI design, especially UMI design VI, has been benchmarked in the hot-spot mutational panel. However, its value in real applications is unclear. Although the authors highlight the improved sensitivity of the structured UMI design, testing the new UMI design in a biological background to identify rare mutations (e.g., with variant allele frequencies below 0.1%) would add substantial value to this method-centric study.

2. The authors emphasize the importance of UMI design diversity based on the UMI collision principle, which is reasonable. However, it is counterintuitive that some UMI designs in the test group I-XIV have low diversity. For example, UMI designs II, III, IX, X, and XIII show lower or equal diversity compared to UMI design III, which could be filtered out based on UMI diversity without further tests. Additionally, in response to comment 2, the authors argued that extending UMI design III for greater diversity with four extra nucleotides would increase simulated UMI-UMI interactions. Given that UMI design III already shows the highest simulated UMI diversity and superior specificity, the correlation between simulated UMI diversity and true experimental performance remains questionable. Therefore, it remains crucial to refine UMI III to enhance complexity and optimize detection efficiency.

**Authors’ response to reviewers**

Reviewer #1: The authors have satisfactorally adressed my concerns.

Reviewer #2: We are pleased to see the improvements in this manuscript, particularly the addition of Supplementary Figures 1 and 2, which clarify the rationale behind the UMI design. The inclusion of a step-by-step guideline (Supplementary Figure 16) and computational tools to simulate the probability of unintended UMIs are also valuable additions. Despite these efforts, the revised manuscript provides seemingly limited additional value compared to the original version though. Beyond the benchmarking experiment, the authors should in our opinion demonstrate that the new UMI design can address challenges that cannot be met by current strategies. Without such experimental proof, even if only a proof-of-concept, it remains difficult to assess the significance of this paper and its impact on the field.

*We thank the reviewer for the constructive and useful feedback to improve the manuscript. To address the remaining issues, we have generated several sets of new experimental data to support our conclusions. We have (1) shown that variant allele frequencies < 0.1 % can reliably be detected in standardized control material. Next, (2) we applied our method to detect circulating tumor-DNA (ctDNA) with variant allele frequencies < 0.01% in blood plasma from a patient diagnosed with leiomyosarcoma. Here, we demonstrate the potential of our methods using personalized ctDNA panels, which is an emerging strategy within liquid biopsy analysis. Finally, (3) we evaluated several new UMI designs, confirming that the selected UMI design was the best choice for downstream analysis.*

Major comments:

1. The structured UMI design, especially UMI design VI, has been benchmarked in the hot-spot mutational panel. However, its value in real applications is unclear. Although the authors highlight the improved sensitivity of the structured UMI design, testing the new UMI design in a biological background to identify rare mutations (e.g., with variant allele frequencies below 0.1%) would add substantial value to this method-centric study.

*Indeed, this is a critical aspect and we fully agree about the value to test our new approach in relevant real-life scenario settings, demonstrating the ability to detect variant allele frequencies < 0.1%. To address this, we have performed several additional experiments.*

*First, we used the hot-spot mutation panel to assess 31 different recurrent mutations at three variant allele frequencies (0.1%, 0.025% and 0.01%) using standardized control material. We could detect all mutations for two of the dilutions (0.1% and 0.025%), while 29 of 31 mutations were detectable at the lowest dilution point 0.01% (note that the number of mutated molecules are in the range between 0 and 2 molecules for this dilution, resulting in drop-outs due to the dilution effect). These new data are shown in three sub-plots in Figure 5A and Additional file 1: Supplementary Figure 18. The new results are described in Lines (252-269).*

*To demonstrate the potential of our approach in a clinically relevant application, we analyzed ctDNA in blood plasma collected longitudinally from a patient diagnosed with leiomyosarcoma. Here, we designed and used personalized ctDNA panels to monitor tumor burden, which is one of the intended applications of our method. First, the primary tumor tissue was exome sequenced and based on the identified mutations we designed a patient-specific and personalized ctDNA panel targeting 18 mutations according to the panel design recommendations (Additional file 1: Supplementary Figure 16). The ctDNA panel was initially applied to tumor tissue DNA to confirm all selected mutations. Next, we analyzed 15 blood plasma samples collected during 283 days with palliative chemotherapy treatment. We show that variant allele frequencies < 0.01% were detectable and that ctDNA data add value to routine clinical assessment. These new data are outlined in a new Figure 6 and Additional file 1: Supplementary Figure 17 as well as in a new result section (Lines 305-355).*

*Personalized ctDNA panels have superior clinical sensitivity compared to broad generic panels since they assess a higher number of true mutations without the risk to detect false positives. To our knowledge, essentially all available PCR-based approaches for personalized ctDNA panels, i.e., targeting multiple patient-specific mutations, are only commercially available with minimal insight into the details of method, such as Natera. Furthermore, they are most often only available as a service. Hence, our approach opens new means for research applications and further development. To our knowledge, this is the first data showing that personalized ctDNA panels are suitable for patients diagnosed with leiomysarcoma. The new data are also discussed in a broader context, in relation to the diagnosis and the use of personalized ctDNA panels (Lines 433-443).*

2. The authors emphasize the importance of UMI design diversity based on the UMI collision principle, which is reasonable. However, it is counterintuitive that some UMI designs in the test group I-XIV have low diversity. For example, UMI designs II, III, IX, X, and XIII show lower or equal diversity compared to UMI design III, which could be filtered out based on UMI diversity without further tests. Additionally, in response to comment 2, the authors argued that extending UMI design III for greater diversity with four extra nucleotides would increase simulated UMI-UMI interactions. Given that UMI design III already shows the highest simulated UMI diversity and superior specificity, the correlation between simulated UMI diversity and true experimental performance remains questionable. Therefore, it remains crucial to refine UMI III to enhance complexity and optimize detection efficiency.

*Indeed, we agree that only stating that some of the UMI designs are not relevant due to low UMI diversity may be counterintuitive without demonstrating additional experimental evidence.*

*To determine how structures related to UMI design III with full diversity performed, we designed four new UMI designs (now UMI design IV-VIII, where V-VIII have full diversity). Our data show that all new UMI designs performed worse compared with UMI design III and thus also worse than our selected UMI design VI (now UMI design X in the revised manuscript) for downstream analysis. However, they all demonstrate improved performance relative to the unstructured reference UMI. Hence, we confirm that UMI design VI (now UMI design X with the updated nomenclature) is the preferred choice in our setup.*

*Related, we also designed a new version of old UMI design X (now UMI design XIV) that also had lower diversity and performed well in the screening. Again, the new and longer UMI design XV with full diversity performed worse than the original UMI design XIV with lower diversity.*

*We have updated Figure 1D with the new UMI designs. The new screening data are added to the plots in Figures 2C-E as well as in Additional file: Supplementary Figures 4 and 6. The text in Result (Mainly lines 108-144 and 155-177) and Discussion (Lines 385-391) are also updated with this new information. We have also pointed out that we have small but noticeable effects of UMI diversity for closely related UMI designs in relation to assay performance, where designs with lower UMI diversity generally perform better (design III versus V, design XV versus XVI and design XVII versus XVIII).*

*To further compare old UMI design VI and UMI design III (design X and III, respectively, with the new nomenclature) in more detail we evaluated 18 additional assays (Figure below). Here, 13 of 18 assays performed better with UMI design X, using quantitative PCR, and 12 of 18 assays performed better with UMI design X using parallel capillary electrophoresis. However, the differences were not statistically significant. In conclusion, data show that UMI design X and III are very similar in performance but X is preferred due to the higher UMI diversity. We have not included these data in the manuscript, but we are open to add them as supplement if recommended.*

***Comparison between UMI design X and III.*** *Eighteen single-plexes were analyzed with UMI design X and UMI design III using 20 ng DNA. (A) Relative specificity using quantitative PCR. Mean ΔCq was calculated as the difference in cycle of quantification values between samples with DNA (PTC) and no template control (NTC). Mean value for each single-plex is shown, n = 3. Box plots of all mean values are shown to the right. n.s., not significant, Wilcoxon signed-rank test, n = 18. (B) Specificity based on correct library product formation using parallel capillary electrophoresis. The percentage of specific library products relative total DNA amount is shown, n = 1. Box plots are shown to the right. n.s., not significant, Wilcoxon*

**Third round of review**

**Reviewer 2**

The authors have adequately addressed our concerns by incorporating three new pieces of work:
1. Expanded UMI Designs: The authors introduced four new UMI designs related to UMI design III with full diversity, along with a new version of the former UMI design X. They confirmed that UMI design VI—now referred to as UMI design X under the updated nomenclature—still stands out in their revised setup, which now includes a total of 19 UMI designs.
2. Testing on Standardized Control Material with Low VAF: The authors tested the selected UMI design on a hot-spot mutation panel containing 31 different recurrent mutations at three different VAFs: 0.1%, 0.025%, and 0.01%. They succesfully demonstrated that VAFs less than 0.1% can be reliably detected.
3. Case Study: The authors presented a compelling case study in which they applied SiMSen-Seq to longitudinally detect circulating tumor DNA (ctDNA) with low VAF (<0.01%) in blood plasma from a patient diagnosed with leiomyosarcoma. This showcases the strong potential of their method in clinical diagnostics using personalized ctDNA panels.

We congratulate the authors on these achievements.
